# Bibliology; or Analytical Reviews of Medical Publications, Interwoven with Original Observations, and Practical Researches

**Published:** 1818-07-01

**Authors:** 


### Second Department.

### BIBLIOLOGY;

OR

### ANALYTICAL REVIEWS OF MEDICAL PUBLICATIONS,

*Interwoven with*

ORIGINAL OBSERVATIONS, AND PRACTICAL RESEARCHES.

Nec tibi quid liceat sed quid fecisse decebit

Occurrat, mentemque domat respectus honesti.

CLAUD.

### Medicine.

### I.

*Practical Illustrations of Scarlet Fever, Measles, Pulmonary Consumption, and Chronic Diseases; with Remarks on Sulphureous Waters.* By JOHN ARMSTRONG, M. D. 1 vol. 8vo. pp. 449. London, 1818.

"The end of every thing should direct the means."

WILLIAM PENN.

Dr. ARMSTRONG, by his Illustrations of Typhus, has insured attention to every future publication from the same pen. We believe that no conscientious practitioner, who is acquainted with this author's writings, can fail to acknowledge the personal obligation and debt which he owes to a man who has so successfully tended to elucidate the pathology, and improve the therapeutics, of some of the most destructive forms of disease which he has to encounter in the weighty and anxious discharge of his professional duties. The public opinion of Dr. Armstrong's late work is sufficiently manifested by the exhaustion of a large impression, and the appearance of a new edition, greatly within the usual cycle round which medical works are, in general, destined to revolve, ere they change their *types*, or receive a fresh momentum from that powerful engine—the press. Dr. Armstrong here sustains the high character which he had so justly earned, in his preceding publication, continuing to combine the patient observation and accurate delineation of Sydenham with the ingenuity of Darwin, and the decision of Jackson.

The present work embraces three important items in the grand class of febrile diseases which our author has undertaken to elucidate; namely, scarlet fever, measles, and phthisis; to which is appended a dissertation on chronic diseases, and sulphureous waters. The value of a work is, in general, proportionate to the difficulty of analysing it. Accustomed as we have been, for many years past, to analytical concentration; and studious as we are of adopting every term and mode of expression, which may convey the greatest quantum of matter in the least possible space, we hesitate not to acknowledge, that to give any thing like a clear view of Dr. Armstrong's volume, now on our table, would require nearly as many pages as the original work! Yet we shall attempt a sketch of this masterly performance; and faint and imperfect as it must necessarily be, it will more than prove that we do not overrate the merits, while we endeavour to appreciate the utility of a volume which, (to use the words of Dr. Armstrong, respecting the works of Abernethy) " we hope will last as long as books last."

### SCARLATINA.

Although Dr. A. with reason, objects to the propriety of the terms scarlatina simplex, anginosa, and maligna, as often conveying erroneous ideas, he adheres to the received nomenclature, rather than introduce innovation. The symptomatology of these three forms is drawn up with all that accuracy of discrimination which characterises the descriptions of Sydenham, and with all the analytical transparency of the French school, untinctured by its peculiar doctrines.

The scarlatina maligna he subdivides into three varieties: the highly inflammatory, the highly congestive, and an intermediate form allied to both—" because it is attended by venous congestions of the viscera, and by a partial and impeded arterial reaction." The delineation of these three varieties surpasses any thing in Dr. A's former work; and is one of the finest specimens of symptomatology which we have ever perused in the English or in any other language. We can only combine a few prominent features.

1. *The highly inflammatory variety,* commences with rigors, dejection of spirits, pain in the head and back, giddiness, vomiting. Sensations of chilliness and heat alternate till the stage of excitement is developed, when the efflorescence appears. Then the fever is intense, and runs an impetuous course. Specks are soon visible on the fauces, at first of a whitish, afterwards of a dingy ash, and lastly of a brown or blackish colour. But in the very formidable cases, death takes place before the throat passes through these gradations; and, in general, it is only when the fever is lengthened beyond the fourth day that there are ill conditioned sloughs, with acrid discharge from the nostrils.

Soon after the stage of excitement arises, the pulse is rapid and vibratory, with determination to the brain, evinced by red eyes, intolerantia lucis, throbbing of the head, delirium, &c. To these succeeds often an overpowering yet imperfect stupor, now and then broken by screaming or fits of fretfulness. In some cases, the abdomen is the seat of pain and heat, with tension, short, anxious breathing, very rapid pulse; irritability and flatulency of the stomach. In others, the pulmonary system is the oppressed point. But whatever region may be principally engaged, excitement soon veers round to collapse, with diminution of heat, failure of the general powers, weakness and rapidity of pulse, laxity of surface, labour of respiration; in a word, those symptoms called putrid and malignant, are now conspicuous. In this stage convulsions, vomiting, or suffocation, according to the organ chiefly affected, terminate the scene. "But in whatever mode death approaches, there is always in the beginning of the disease, a marked and unequivocal stage of high and general excitement, to which the appearance of extreme debility and putrescency of the last stage may be clearly traced as mere consequences." p. 11. This we conceive to be sound pathology.

Although in the short stage of oppression, which precedes the developement of excitement, there is always more or less of venous congestion of the internal parts; yet it is in the second stage that the main mischief is generally produced, both in the s. malig. and anguinosa, which, indeed, differ only in the grade of excitement.—"From a cautious survey of the symptoms during life, and the examination of several bodies after death, I am warranted in affirming, that the brain, the liver, the stomach, the intestines, and the lungs are the parts most often inflamed." p. 14.

2. *Irregular congestive Form.*—Here the reaction is partially or irregularly developed; and though not so dangerous or protracted as the next variety, yet often proves fatal from bad management, or the peculiarity of its nature. This species is drawn in a masterly manner by our author, and defies analysis.

3. *Regular congestive Scarlatina.*—Here the attack is generally sudden, with paleness, faintness, and sickness; pain, load, or giddiness of head; extreme oppression; uneasiness in the region of the heart, or at the pit of the stomach. The attack once formed, the respiration is quick and anxious, or slow and impeded. The mind at first confused or dejected, soon becomes delirious, succeeded by stupor, under which the patient finally expires. The pulse from the beginning is low, quick, and irregular. In some cases, where nature attempts a feeble reaction, the pulse feels rather sharp for a certain period, ultimately growing weak and undulating. Bowels flatulent, constipated, or irregular in the first stage; frequently loose in the last. Stomach irritable in general, sometimes retentive. This form runs its fatal course in two, three, or four days from the occurrence of extreme depression, accompanied at last with symptoms of putridity. In this form the efflorescence is from the first of a purplish or copperish hue, which deepens as the disease advances—sometimes quickly receding and never returning. When the disease continues beyond the fourth day, there are generally specks or sloughs in some part of the fauces. Dr. A. does not consider the affection of the throat as the cause of death in any species of scarlatina. In *this* it is to be sought "in venous congestions of the brain, liver, spleen, lungs, or the vessels about the heart." The signs of putridity or malignancy do not, our author justly asserts, constitute a primary or essential part of this form, but are purely the consequences of excessive congestions; for if we can remove *these* we prevent the others.

Dr. A. observes, and well observes, that in the open forms of fever, where heat and arterial reaction are clearly developed, the danger is to be estimated by the degree of general excitement, and local determination; whereas, in the masked or congestive forms, the danger is proportioned to the *defect* of excitement, and to the extent of the local accumulations of venous blood. These last have not been sufficiently attended to in this country—physicians keeping their eye too exclusively directed to the phenomena of the *arterial* system, though though it is in the *venous* that we must look for the foundation of many important diseases. In the following pathological observations we fully coincide, as they are extremely consonant with the views which we have long taken of the ratio symptomatum in febrile affections.

" One of the first obvious operations of contagion, like that of cold, is a change of action in the cutaneous vessels, and a recoil of blood from the surface towards the centre. But contagion produces also, directly or indirectly, a peculiar effect on the nervous system, which is chiefly evinced by the disturbed functions of the brain and of the stomach. The change of action in the cutaneous vessels, the recoil of blood towards the centre, the want of sensorial powers, and the disorder of the stomach, all concur to oppress the heart and the arteries; and we accordingly find, that the arterial circle every where beats r. ore languidly than natural, a superabundance of blood being accumulated in the larger internal veins. Now this venous accumulation is sometimes so great as to overwhelm the functions of the brain, lungs, liver, or heart; and the last-mentioned organ collapsing, the usual interchanges of blood between the arterial and venous apparatus are carried on no longer, and the death of the whole system necessarily ensues. In general, however, the venous congestion is not thus mortally overpowering at once, but rather of such inferior degrees as to rouse at least into some resistance the latent energies of the heart; and indeed it most frequently happens, that a tone is at length acquired, which gradually extends throughout the whole arterial circle. The stage of reaction, therefore, whatever may be its final tendency, is immediately beneficial. Whenever the stage of reaction is not partially or perfectly developed in febrile diseases, the danger is instant and imminent. Most sudden deaths arise from great venous congestions. It cannot be doubted, that venous congestions are, *cæteris paribus*, more dangerous than arterial reactions; because, as the former are more rapid, and also more difficult to remove, so the latter relatively give longer time and greater facility for medical aid; nay, sometimes spontaneously subside into health. Arterial reaction is the mean by which Nature preserves the human frame from the sudden and dangerous shocks of venous congestion." P. 30.

---

*Treatment.*

It is, then, to the different degrees of excitement, and of congestion, that all the modifications of scarlatina owe their pathological peculiarities, and hence the rules of treatment must be as various as the characters of the diseae. These rules are laid down by our author with all that lucid arrangement and accuracy of discrimination which characterize his Illustrations of Typhus, and which we are convinced every zealous cultivator of the healing art will make himself master of, not merely by a perusal, but by reiterated study of the work itself.

In that stage of oppression which usually precedes reaction or arterial excitement, much may be done by watching the nascent movements of nature, and assisting her salutary efforts. Thus, when the contagious principle of scarlatina begins to unfold itself by paleness, headache, sickness, and lassitude, a brisk purgative, a mild emetic, and the tepid bath, *in succession*, are often of essential service by equalizing the circulation, and rendering the future case milder and more manageable. Even in this stage of venous congestion, if the head labours much, leeches to the temples, or a *little* blood from the arm, *after* the tepid salt water bath, will generally give relief and moderate the succeeding excitement.

When the reaction is fairly emerged, in the simple scarlatina, *tepid* affusions four or five times in the twenty-four hours, an active aperient daily, rest, ventilation, cleanliness, and a bland, liquid diet, will in general be sufficient, with light animal broths, in the subsequent stage of collapse.

In the scarlatina anginosa, the same measures are to be pursued in the first stage; but when the excitement is manifested by a morbid heat and dryness of the whole surface, the *cold* affusion is to be promptly employed, and repeated, *pro re nata*. Dr. A. thinks that *this* measure is seldom useful after the third day of the excitement, and we fully agree with him, that the efficacy of cold affusion *alone* has been much overrated both by Dr. Currie and his followers. Cathartics ought always to be employed at the same time, and contrary to the usual routine, they should be made to act in the night as well as in the day. Indeed, they are more serviceable in the *former* than in the *latter* period, for it is the period of exacerbation in all pyrexial diseases.

When the above measures do not afford relief, we may be sure that some latent inflammation exists, and venesection must be early employed. But when there are any appearances of approaching collapse, or gangrene in the throat, the lancet must not be used.

"In no febrile disease do large doses of calomel seem of more decided benefit than in this, when daily determined to the bowels by some other auxiliaries. Confident in this important fact, by numerous observations, I have not hesitated to prescribe calomel boldly throughout the whole stage of excitement, always accelerating its operation by jalap or rhubarb, with moderate doses of sulphate of magnesia; and it is somewhat remarkable, that though I have given six or eight grains of this mercurial to children, twice, thrice, or even four times in the twenty-four hours, yet I have not once seen ptyalism supervene." P. 50.

This accords with our own experience.

"It is still (says this accurate observer) too much the fashion to declaim against the bold administration of calomel in the commencement and progress of ardent fevers. Only let practitioners put calomel fairly and extensively to the test in febrile diseases, and we shall soon cease to have imaginary and unfounded clamours against its free employment." P. 51.

In the inflammatory form of the scarlatina maligna, if the excitement be not met by the most prompt and decisive measures, the lesion of some important part will be the consequence, and death the result. Dr. A. like most others, we believe, who speak candidly, found bark, wine, and aromatic cordials, most pernicious in the first stage, and destructive in the second. The reaction must be met by the cold affusion; and if that should not reduce the fever, a vein must be opened in the arm or neck, or a branch of the temporal artery, and the blood allowed to flow till faintness approaches. The rules laid down in typhus are here applicable; but as the excitement runs higher, the first bleeding must be a most decisive one, so as to induce a change of action in the whole of the circulatory system. If a second venesection should be deemed inadvisable, and yet relief not obtained, leeches are to be applied to the temples or the region of the liver. The head is to be raised and refrigerated. The bowels are next to be acted upon by large doses of calomel and jalap, aided by the neutral salts, and the purgative plan must be persisted in till there is a visible change for the better.

It need hardly be stated, that this active system of depletion is limited to the stage of excitement, and in general, to the first thirty hours of that stage; if persevered in later, it can only hurry on the fatal termination. We cannot follow Dr. Armstrong through his minute and inimitable instructions on all points of management, but refer to the work itself. He declaims with justice against the erroneous notion of nursing a febrile patient in the beginning, to enable him to meet the collapse at the close. The reduction of excitement is the best cordial, and the best preservative of strength; and where this is early and decisively effected, the collapse is proportionally manageable.

In the irregular form of the congestive scarlatina, purgatives and the warm bath are the best remedies; especially if early employed. The bath about once in twelve hours; the purgatives so as to procure about four or five stools daily. The appearance of the stools here indicates a derangement of functions in the liver, and therefore calomel should be freely administered, and then determined to the bowels by cathartics.

As the attack of *regular congestive scarlatina* is generally open, sudden, and overpowering, the period for venesection is commonly limited to the first twenty-four hours of the decided signs of the congestion. An universal collapse soon supervenes, and the mass of blood is changed into a dark, uncoagulable gore. The first thing to be done is to immerse the patient in a warm salt-water bath; and while he remains in it, or immediately on coming out, to abstract blood till the pulse is free from oppression. When the warm bath is not at hand, frictions by warm flannels and warm stimulating liquids must be used instead; and bladders of warm water applied to the feet and other parts. These measures bring back the recoil of blood, and relieve the visceral congestions. Bleeding is here to be more cautiously practised than in the other forms of the disease. The finger must be kept on the pulse. If it rises during the flow, we may safely proceed to a moderate extent, and *vice versa.* Venesection should never be carried to syncope in great venous congestions. When there is free return of blood to the skin after venesection, warm diluent drinks are to be administered; but if the skin remains cool, a little warm wine and water may be given, until some degree of arterial excitement succeed. Immediately after the use of the warm bath, frictions and bleeding, a large stimulating cathartic enema should be injected. Calomel is then to be given boldly, so as to create a free, warm, and universal perspiration, with a copious flow of bile. These are sure to equalize the circulation.

"In the most formidable examples," says Dr. A. "of the highly congestive variety of scarlatina, the secretion of bile is often totally suspended at the commencement; the liver being in a state precisely analogous to that which occurs in the worst forms of *Cholera Morbus*, so gorged with venous blood as to be incompetent to perform its wonted offices. All the instances of this modification of the scarlet fever which proved rapidly mortal, were designated by an absence of bile in the motions; and the same has been the case in all the most concentrated attacks of *Cholera Morbus* which have fallen under my inspection." 87.

This was formerly heterodox doctrine, and has yet but a few ostensible supporters. It may, nevertheless, be founded in truth, that, "What every body says must be true," is an adage which makes but a poor figure in the annals of the healing art! We would recommend the following passage to the serious perusal of those who contend that mercury is inert or injurious in all dangerous fevers.

"In ordinary complaints, ptyalism is one of the *first obvious effects* of mercury, but it is often the *last* in those of an extraordinary nature; hence, in the highly congestive scarlet fever, we most frequently find the liver, kidneys, and skin excited by the calomel, *while the salivary glands remain unaffected.*" 88.

Upon this circumstance, we suspect, has been founded the erroneous opinion that mercury is inert till the fever is over, when it shews its specific effects.

"Mere salivation," says this distinguished Physician, "is at no time to be considered as the principal part of the efficacy of mercury, yet it is one of the surest signs that it is operating on all the secretory organs of the body; the conjoint evacuations elicited from them, and the return of the tide of the circulation into the arteries, being the means by which the *local engorgements* and the general oppression are relieved in congestive diseases." 89.

Several most interesting cases, illustrative of our author's practice, are chiefly related, and to these we refer. The Doctor makes many admirable observations on our conduct during the convalescent stage. For some time after the removal of the congestions, there is a considerable relaxation of the general habit, "combined with an irregular disposition to arterial excitement; and any canse which increases this disposition may occasion an internal or external dropsy, as the absorbent system seems unequal to counterbalance any excess of the secretions." "In every point of view then, the common practice of administering excitants during convalescence is most exceptionable." This is consonant with our own experience. After every acute disease there is a rapid disposition to make blood from every material, and unless guarded against, a relapse is often the consequence of a weakened organ giving way.

Imperfect as this sketch is, it has carried us so far forward, and beyond our proportionate boundaries, that we shall be forced to pass entirely over those important divisions of Dr. Armstrong's work, which are occupied with the subjects of measles and pulmonary consumption. We could do nothing like justice to the author in attempting to analyse such exquisite specimens of symptomatology, pathology, and therapeutics; and, moreover, Dr. Armstrong's works need only to be announced, to insure professional perusal. The concluding division, however, of this invaluable volume is so singularly interesting, that we shall dwell upon it, even beyond the prescribed limits of this article.

### CHRONIC DISEASES.

Every one knows how *much* under controul are acute, and how *little* are chronic diseases! Want of general success in the *former* must be the practitioner's own fault, provided he be early called in; for the united agency of blood-letting, purgatives, mercurials, blisters, and opium, will commonly subdue the very elements of these disorders, when judiciously and opportunely administered. We cannot say the same of chronic ailments. This seems partly owing to the obstinacy of their nature, and the vagueness of opinion respecting them. Dr. A. for more than ten years past, has directed his attention to this extensive class of human afflictions; and means, should health be spared, to dedicate a large volume to their history and treatment. Mean time the following brief results of his observations are offered to professional consideration.

Between chronic and acute diseases, Dr. A. justly remarks, a greater affinity exists than is imagined. The first stage of both consists in *functional,* the last in *structural* derangement. If high degrees of venous or arterial turgescence mark acute, low degrees of the same attend chronic complaints.

Chronic *venous* congestion generally occurs in the brain, liver, spleen, or vicinity of the heart.

In the *brain,* it causes uneasiness of some kind in the head, with disturbance in some of the external senses, and irregularity in the periods of watching and repose. To these are added mental and corporeal languor; forgetfulness; pallor of the face and whole surface; languid or oppressed pulse; diminished or irregular temperature of the skin; increased susceptibility of aerial transitions; weakness in some of the members, occasionally accompanied by wandering pains.

This venous congestion of the brain is often conjoined with a similar affection of the *liver,* the symptoms of which are obscure for a long time. Fulness or uneasiness in the right side on motion or pressure denotes hepatic affection easily enough, but these symptoms are often wanting, and the stools, therefore, should be carefully examined, as well as the appearance and functions of the skin and urinary apparatus. Gastric, and what are termed nervous affections, are very commonly dependent on venous plethora of the hepatic system or the brain, or on both combined.

Chronic venous congestions of the *spleen* are generally marked by uneasiness under the ribs of the left side, with cough, indigestion, and nervous feeling.

When the large veins in the vicinity of the heart are congested, palpitations, with weight and anxiety, are felt in the cardiac region, attended by sudden attacks of giddiness, faintness, or nausea, with much irregularity of pulse; indeed, chronic congestion in any part of the nervous system is always coupled with disordered action of the heart.

Venous congestion determining chronic disease, generally occasions in the end, excitement of the heart and arteries, but in a very irregular manner, so that health is suspended between alternate collapse and fever. When arterial excitement is permanently established, the venous congestion may be said to terminate in a new disease. Indeed, venous congestion is *naturally* cured by increased action of the heart and arteries, or by some increased secretion, particularly of urine, perspiration, or pulmonary halitus; a fact which physiologists have hitherto overlooked, if we except Dr. Parry, who gives various intimations of these salutary movements, in his Elements of Pathology and Therapeutics.

Dr. Armstrong has here detailed numerous interesting practical observations on the abuses of malt and spirituous potations, intemperance in food, the injudicious use of opium, calomel, &c. for which we refer to the work itself. His Philippic against *tonics* ought to be inserted in every dispensatory and pharmacopœia in the kingdom, in order that bane and antidote might go together. "If these (tonics) were erased from the pharmacopœia, it would be a real benefit to the profession and mankind, for they only serve to mislead the former, and to tantalise or injure the latter." 384.

The symptoms which characterize chronic *inflammations* of the various organs and internal tissues are next entered upon by our author, and handled with all that ability which he has shewn in the delineation of acute diseases. We offer the following passage, because it involves important doctrines, which we shall, in a future number of this Journal, discuss; and because it coroborates the sentiments of Dr. Philip relative to the state of the vessels in inflammation; sentiments which we have always considered as highly deserving the attention of the profession.

"In diseases of chronic *congestion* there is preternatural fulness of the internal veins; in chronic *inflammation* there is preternatural *fulness* of some part of the capillary *arteries;* and this fulness is so great as to disturb the functions of the organ in which it exists, and the whole economy by consequence is thrown into disorder. But the topical fulness, whether seated in the veins or in the arteries, may originate in general causes which give a shock to the heart, the main spring of the circulation; and in such cases, the seat of the topical disease is determined by the predisposed states, or by the weakness of the respective organs attacked. If there be any predisposition to disease in an organ, that disease may be readily excited by any thing which greatly disturbs the action of the heart; for though the predisposition remained latent when the whole circulation flowed with its natural calmness, yet when that circulation has once been agitated by some shock at its source, every organ must participate in the change, but especially the weakest. On the other hand, if any disturbance should take place in the circulation of a part, the currents of blood sent by the natural action of the heart, if they should not remove, must necessarily increase that disturbance, upon the known principle of the motion of fluids; unless indeed those currents find other adjacent channels of conveyance, which are furnished by anastomoses, seemingly to provide for the prevention and reparation of injuries. *What has been called increased action of the arteries of a particular part, is really a state of those vessels very different from increased action: for the action of one artery cannot be increased more than that of another independent of the heart; and what seems to be so, is obstruction or distension, augmented by the natural or preternatural action of the heart, as shall afterwards be shewn.* In conformity to the view above given, we lessen the action of the heart by evacuations, whenever we wish to remove a local disease affecting an important organ. If any local disease should finally be so great as to increase the action of the heart by creating a nervous irritation, other organs which had been previously predisposed may become disordered by that increase of action, in which the whole arterial circle participates. Thus, in chronic as well as in acute diseases, the heart is directly and indirectly concerned in their origin or progress; and much of what is denominated sympathy may be traced to a direct disturbance of the heart from some constitutional shock, or to an indirect disturbance from some topical disorder." 396.

In Dr. Armstrong's work on typhus, an attempt was made to shew that a combination of calomel and opium, after bleeding and purging, had a strong tendency to restore the balance between the venous and arterial systems, by rousing the heart into play, and thus removing acute and sudden congestions. Dr. A. thinks that the same combination is often useful in chronic congestions, especially if the liver be affected; but mercury here should only be given to correct the alvine evacuations, and excite the heart and secretory organs. When these purposes are answered, it ought to be wholly withdrawn.

"While short courses of mercury may be beneficial, long ones often break up the general strength, and leave the nervous system in an irritable, and the vascular in a most vacillating condition." 402.

Dr. Armstrong observes, and in this observation we coincide most fully, that "many of the chronic inflammations of the viscera may be traced to the influence of our variable atmosphere on the surface, and from thence *sympathetically* on the various organs of the interior." P. 405.—The coincidence of ideas between Dr. Armstrong and a recent writer on this subject, is surprising; but as there was no communication, it is strongly in favour of the truth of the doctrine.

In chronic inflammations of internal organs the treatment is to be regulated, and yet on general principles.

"Most frequently, small or moderate venesections should be first tried from the larger veins; and these should be followed up by local bleeding, blistering, purgatives, rest, and an antiphlogistic regimen: but where success does not attend these measures, small doses of calomel or the blue pill may be prescribed until the system be slightly affected, and then, for the most part, they should be suspended, and the laxative plan continued." P. 406.

But the most interesting, we had almost said surprising part of the work yet remains to be noticed; for if one half of Dr. Armstrong's observations be confirmed by the experience of others, (and he is the last man whom we would suspect of exaggeration), a total revolution will, ere long, take place in the current of invalids toward medicinal springs.

So far back as the year 1807, Dr. A. was in the habit of recommending patients affected with chronic visceral inflammations and congestions, to drink the sulphureous waters of HARROWGATE, merely with the hope that their purgative qualities, combined with change of scene and air, might possibly be effective in a class of complaints which too often baffle the physician, and ultimately destroy the patient. Finding them remove an hepatic complaint which resisted every other remedy, his attention has been directed to the operation of Harrowgate, and also of similar sulphureous waters, so that the "following remarks may be considered as the result of pretty extensive experience."

The first thing that forcibly struck our author was this, that the continued use of these purgative waters, for days and weeks, produced no debility; on the contrary, the patients gained both strength and flesh, notwithstanding they had daily and copious evacuations. Dr. A. was naturally led to conclude, that the salutary effects of these waters resulted from their aperient, and yet non-debilitating attributes. But chronic diseases having fallen under his observations, at various times, in which the said waters were most decidedly beneficial, and that too when the bowels had been but scantily moved, he was led to inquire whether they were not endued with some other powers that had escaped his notice. In attending more closely to their operation, he found that they "acted most powerfully on all the secretory organs of the body, but more especially on the liver, on the kidnies, on the villous coat of the intestines, and on the skin." P. 414.

Subsequent investigation convinced our author, that the sulphureous waters of Harrowgate and Dinsdale had a direct influence over chronic inflammation, wherever seated—whether on the surface or in the viscera, and that this power depended on the sulphurated hydrogen gas which they contained. Dr. A. observes, that the most enlightened practitioners of the present day attribute the powerful alterative effects of mercury in visceral diseases to its action on the secretory organs.

"Now, in the sulphurated hydrogen gas, (says he) we have another agent which acts as powerfully as mercury on the secretory organs; but with this difference, that while the long continued use of the latter in chronic diseases, generally breaks up the strength, that of the former generally renovates the system. The sulphur and hydrogen gas, then, has a decided superiority over mercury in chronic diseases in general; and this is not a speculative opinion, for I have proved its correctness in numerous instances, by the test of experience." P. 416.

It certainly would be a desideratum in chronic diseases, to possess an agent that should operate *daily and favour-*

*ably* during the long period which is required to eradicate obstructions that had slowly advanced, and that without injuring the general system. If Dr. Armstrong then has really ascertained their powers in this respect (and we do not question his discrimination) he will have conferred a greater benefit on mankind than Jenner.

Dr. A. brings forward other instances of the analogy between the action of sulphurated hydrogen gas and that of mercury. It is now well known, that when the system resists the specific action of mercury, it is a certain test that a high inflammatory diathesis exists, which is the cause of the resistance; for lessen this inflammatory diathesis, and the specific action will be readily induced. The same holds good in respect to the action of sulphurated hydrogen gas.

"During a series of years, (says our author) I have traced the operation of the sulphurated hydrogen gas from one organ of the body to another; from the skin, joints, and eyes, to the viscera of the head, chest, and belly: and the sum of my observation authorises me to declare, that it is one of the most powerful antiphlogistic agents which can be found; for wherever the chronic inflammation be seated, it will more frequently remove it, than any other single expedient which has hitherto been used and recommended by the medical faculty. P. 419.

Dr. A. thinks, that even in chronic affections of the liver, the medicine in question is superior to mercury. "In many hepatic diseases, however, it will be best to combine the operation of these two remedies at the same time."

"Small doses of calomel, or of the blue pill, may be given for a short period every night at bed-time; and a large tumbler glassful of the Harrowgate water may be administered on the following morning before breakfast, and repeated every twenty minutes until it operate by the bowels. But in such cases, the mercury should not often be given longer than for a week or ten days; some mild purgative pill may then be substituted at bed-time, and the sulphureous water continued every morning until the symptoms entirely disappear." P. 420.

In what are called stomach complaints, Dr. A. thinks that a regular perseverance in the Harrowgate waters will frequently do more good than all the medicines in the Pharmacopœia. "But in these, as well as in hepatic affections, it will sometimes be requisite to assist its purgative operation by an occasional blue pill, or by one of the compound rhubarb or aloetic pills at bed-time." P. 420.

Chronic rheumatism, gout, and almost all cutaneous affections, will yield more rapidly to the continued internal exhibition of the sulphurated hydrogen gas, Dr. A. avers, than to any of the means now commonly employed. But in these, as also in most chronic complaints of the viscera, the recovery will be considerably expedited by the frequent use of *tepid baths,* which contain the sulphurated hydrogen gas.

Although Dr. Armstrong's experience of this remedy in *pulmonary phthisis* is not yet sufficient to enable him to say much on the subject,

"Yet it has seemed exceedingly useful in several instances where phthisis was distinctly threatened, especially when the pectoral symptoms were complicated with hepatic disorder, as frequently occurs. In a few solitary cases, which bore the characters of genuine and confirmed phthisis, *and in which pus was expectorated,* a marked change for the better took place from the drinking these waters; *and I recently saw two most remarkable examples, which appeared to be actually cured by the mineral spring at Dinsdale, though in both cases the disease was far advanced when it was first tried.*" 422.

Now, as all the measures hitherto tried for the cure of phthisis have failed, not even excepting the fumes of tar, it becomes a question of the greatest importance to society, that the effects of sulphurated hydrogen gas, as combined in the Harrowgate waters, should have a fair trial, since the fact of two cases being cured under the inspection of such a man as Dr. Armstrong, offers a more cheering prospect to the healing art than has been presented for many years. Dr. A. thinks that artificial waters possessing the properties of the Harrowgate and Dinsdale springs might be easily prepared, and proposes that a trial should be given to them in some of the public hospitals—a proposition which we trust will be put into execution by some of the able and zealous medical officers of these Institutions, both in the metropolis and elsewhere.

At the conclusion of the work, Dr. Armstrong gives us a rapid outline of an intended volume on "the common excitive fever, the disorders which precede and produce water on the brain, and the generalization of the subject of all acute fevers."

From this outline we shall make one more extract, and then conclude our analysis.

"The common excitive fever most frequently originates from the influence of the weather, but it may also proceed from intemperance, irregularity in diet, fatigue of body, anxiety of mind, and other causes: and when the brain is the organ chiefly affected, it is liable to be considered as phrenitis, but more often as genuine typhus, though it is never contagious; yet as the lungs, liver, and intestines, may be separately or combinedly attacked in it, so its name might be, and has been varied, according to the seat of the most decided disturbance. But by a reference to familiar and unquestionable facts, I shall be able to shew, whenever the common excitive fever is ushered in by a cold stage, that both the pyrexial and the inflammatory symptoms are the effects of a general excitement, which arises out of the *venous* congestions of the cold stage; and consequently, that neither increased heat, nor the arterial disturbance called inflammation, are, in such cases, any part of the original train of morbid phenomena. It shall be further shewn, that the most decided seat of the topical inflammation, which occurs in the general excitement, is determined by the particular states or predispositions of the respective organs on the occurrence of the excitement. So that in one patient the brain may be inflamed, in another the lungs, in a third the stomach, and so forth, agreeably to the previous conditions of those organs; and yet the nature of the disease in all these cases is essentially the same, its main force having only been spent upon different organs, from incidental circumstances. This view of the common excitive fever will strike at the very foundation of that doctrine which makes fever always an effect of topical inflammation: since it will be proved, that both the fever and inflammation alternately arise, as consequences, out of preceding actions, which mark a deficiency of heat and of arterial reaction." P. 447.

---

We need hardly recapitulate our sentiments respecting Dr. Armstrong's volume, after what has already been said. It is only inferior to the work on Typhus, because, in that work, Dr. Armstrong has laid down the great fundamental points of pathology; which, in subsequent discussions relative to febrile diseases, can be little more than repeated. We conceive, however, that this Physician's writings are well calculated to diffuse a beneficial influence over the minds of the Medical Profession at large; and we hope that in the metropolis, where he has now taken up his residence, his opinions and practices will make a deep and salutary impression on the rising generation of students. In London, too, we are inclined to hope that Dr. Armstrong will meet with that kind reception from his brethren which distinguishes the great and liberal members of our profession, and to which his zeal and talents so well entitle him. Our best wishes attend him in all his exertions for the improvement of medical science—in every stage of his journey through the vicissitudes of life!

### II.

### *Observations on the Epidemic Fevers of Dublin, founded on a Report of the Hardwicke Fever Hospital, during the Years* 1813, 1814, *and* 1815.

By Edward Percival, M. D. &c. one of the Senior Physicians to the Hospital, &c.

Although this Report forms but a part of a volume, [the Transactions of the Dublin Association] yet we deem it useful to make it the subject of a separate analysis, both on account of its extent (120 pages) and the value of the matter. A great deal of this paper, however, is taken up with statistical remarks and *local* observations which can only be *locally* interesting; and therefore not necessary to be introduced here. For the sake of brevity, our great object, it must be understood, that we are always speaking the sense of our author, unless we distinctly appear *in propria persona.* This plan saves a prodigious waste of words.

*Contagion.* Dr. Percival discusses the point of the spontaneous or equivocal generation of contagion with much ability and candour. He is aware that some modern writers have endeavoured to disembarrass the science of physic of these questions, by various artificial and hypothetical arrangements; but doubts whether they have, in any respect, invalidated the experience transmitted to them concerning the equivocal generation of typhous fevers. He disagrees, therefore, with Dr. Bancroft on this point, and proves from authentic statements and extensive observation, that almost any fever, however simple in its origin, may *acquire* a contagious character by crowding, filth, imperfect ventilation, &c. as we have all along maintained in the pages of another Journal.*

Dr. P. justly remarks, that the leading features of epidemic and contagious fevers are rapid prostration of strength, with sanguineous determination to the head, or other principal organs, attended with frequent pulse, and suspended or disordered secretions. " They differ from fevers arising from simple local inflammation in many particulars; but in none more remarkable than in the sudden

* Vide our Reviews of Pym, Burnett, Fellowes, &c. in the *Medico-Chirurgical Journal,* Monthly Series.

failure of the mental and voluntary power, and in their uniform tendency to perform a certain cycle of morbid changes, in definite periods." P. 295.

*Typhus Fevers.*—These, preceded by great bodily fatigue and mental anxiety, were uniformly hazardous. The worst symptoms are, pervigilium, tympany, singultus, coma: the best, sleep, a moist tongue, solvent bowels. Deficiency of urine is an unfavourable, suppression a fatal symptoms. The pulse is *res fallacissima.* The countenance and posture of the patient, his manner of *respiration,* and the appearance of the tongue, afford authentic information to the observant practitioner. When the patient lies at ease on his side, and especially when he relieves himself by spontaneous change of position, after the fever is much advanced, the augury is favourable. On the other hand, when he continues extended and supine, lethargic and muttering, the prognosis is bad. But the tongue is the most accurate informant of the state and progress of the fever, as it indicates the condition of the whole intestinal canal, and those viscera which hold so remarkable a sympathy with the brain.

*Duration,* generally fourteen days. *Crisis* was commonly marked. The most favourable was by a profound sleep, often of forty or fifty hours duration. But the critical period was often a scene of severe struggle, the issue of which was, for many hours, doubtful.

*Dissections,* presented, *in typhus, with sub-delirium and comatose affection,* the usual marks of vascular congestion in the brain; effusion of blood under the cranium; the vessels of the pia mater and plexus choroides turgid, and the capillaries gorged with blood; more or less of serous effusion in the ventricles; numerous bloody points on the cut surfaces of the brain.

"In many fatal cases of petechial fever, however, the brain exhibited very slight evidence of sanguineous congestion." "In almost every case, whether of mild or malignant typhus, one or other of the following organs were also found engaged with disease; the lungs, the pleura, the liver, the peritoneum, the mucous or villous texture of the intestinal canal." P. 304.

We should conceive that this is strong evidence, that although fever has no *exclusive* "local habitation," it never fails to commit ravages on some important texture in the living fabric, before it snaps the thread of life. The influence of spirituous liquors on the hepatic system was here exemplified by "the frequent appearance of *diseased liver* among the patients of the Hardwicke Hospital," on account of the number of *decayed drunkards* in the pauper's buildings, which were *then* much visited with petechial fever. A vein of predominance, in the disorganization, was observable in each season. "Thus pulmonic disorganization was chiefly detected in spring; gastric derangement in the summer; hepatic and enteric in the autumn; and cerebral congestion in the winter;" a clear proof that *no one organ* is the *exclusive* property or theatre of febrile derangement.

Dr. Percival truly remarks, that the pathological investigations of modern physicians have done little more than bring us back to the *practice* of Sydenham, who had not the light of morbid anatomy. But he adds,

" Observation having confirmed what analogy suggested, and familiar facts having been authenticated, and, in some degree, explained by organic appearances, the science of fever, though not greatly enlarged, rests undoubtedly on a more solid basis than at any former period." P. 307.

In the following sentiments also we cordially join.

" What has hitherto retarded the advancement of this work, has been not so much a propensity to generalize, which belongs to all philosophic minds, as an eagerness to abandon old doctrines, whenever new ones have been projected, and to rely too exclusively upon each, for a solution of all the various phenomena of fever. Thus the humoral pathologists slighted the Hippocratic precepts concerning morbid temperature; and in turn the humoral theory, though pregnant with the most important doctrines of the economy of secretion, was rejected for neurological hypotheses of debility and spasm. Organic disease was thus in a great measure overlooked, until the doctrines of morbid heat, and local congestion or inflammation again brought this important feature into notice. But there is now danger, that in discharging the cycles and epicycles of fever, delineated by Cullen or Darwin, the doctrines of *morbid catenation, or associate actions of disease* will be, at the same time, fatally overlooked." P. 309.

Some excellent remarks follow on the pulse, the tongue, the petechial eruptions, &c. from which we shall select a few. *Pulse.*—Extreme frequency, fluctuation, and compressibility may result from the presence of acrid secretions and tough mucus in the stomach; the effusion of morbidly concocted bile, or the accumulation of indigested aliment and vitiated humours in the intestinal canal; an erect posture of the body; restlessness; pervigilium; the presence of too much light, heat, or noise in the patient's room.

" The worst kind of pulse in typhus is that which gives the sensation of equable and permanent dilation, with a certain rigidity or wiriness. This phenomenon occurs towards the close of the disease, or when the surface and extremities of the body are cold from defective energy of the heart and arteries. A warm diaphoresis often resolves this muscular rigidity, and restores the undulating character of the pulse."

*The Tongue* may be moist and thinly coated with white mucus, as in febrile phlegmasiæ, where there is usually strong pulse; high temperature, and visceral inflammation. 2dly. Tongue coated with thick yellow mucus; skin suffused with yellow; the epigastrium tumid; hepatic system in a state of congestion. 3dly. A brown dry streak in the middle of the tongue, indicating intestinal torpor and defective secretion throughout the canal. 4thly. A tremulous tongue betrays a debility with sensorial disturbance. 5thly. A dark, dry, and shrunk tongue, with difficulty protruded beyond the teeth, indicating a deeply vitiated condition of the secretory organs, with extreme prostration of animal and vital forces. Although venesection is seldom of use in these cases, a spontaneous hæmorrhage, especially from the nose, is often salutary. A copious exhalation from the capillaries tends to the like relief. 6thly. Tongue of a florid red hue with eminent papillæ; this was chiefly observable where the fever had become hectical from suppurating surfaces or abscesses. 7thly. Tongue swelled and shining, from which no particular indication could be drawn. Upon the whole, the information afforded by the state of the tongue is both various and accurate, and claims our primary attention and reliance.

Petechiæ are often banished in forty-eight hours by the cool and purgative plan of treatment. These cuticular phenomena are never critical; they are cherished and multiplied by foul air, external heat, and internal constipation. The appearance of the petechiæ, with or without fever, is hitherto unexplained. The common theory of inflammation throws as little light on these phenomena as the obscurer doctrine of putrescence.

---

*Treatment.*

Although a considerable number of patients recover from fever without any treatment, yet when we consider the formidable *sequelæ,* incident to these diseases, or the vitiated, and irreparably broken down constitutions which abound in large towns where epidemic fevers chiefly prevail, we shall be satisfied that the mere preservation of life is not the only object of medical skill, nor the only essential point of contrast between the various curative methods recommended by discordant authorities.

"That sceptical indifference which slights the comparative pretensions of dissimilar modes of treatment, even on the ground of their correspondence in some leading features, will be found to arise from want of experience or want of discrimination." Page 318.

The *first* point that is to be attended to, for the cure of typhus fever, and the prevention of contagion, is a plentiful supply of cool and fresh air. This is one of the most powerful auxiliaries in tempering morbid heat, allaying irritation, banishing petechiæ, and promoting sleep and moderate diaphoresis.

"The *next* point to be regarded is suitable *evacuation;* and primarily, that of blood-letting; a remedy that has been viewed with so much exaggerated apprehension on the one hand, and such undue or indiscriminate partiality on the other." 320.

"It is obvious, in every case of epidemic fever, *that the balance of the vascular economy is considerably disturbed;* that the secretions are vitiated or suspended; that partial congestion, or local inflammation obstructs the due exercise of one or several of the more important organs. Thus the skin, the mucous tissue of the primæ viæ, the brain, the liver, and the lungs, are more or less engaged with congestion, and respectively deranged in their functions, according to the constitution of the patient, or the reigning epidemic of the year." P. 321.

This, we conceive, is sound pathology, and is precisely what careful observation suggested to us many years ago, at the bedside of sickness. Dr. Percival's therapeutics are of course excellent, as founded on so secure a basis.

To relieve the oppressed viscera, and restore the lost balance of their functions, *evacuants* of various kinds are the chief remedies. The cold affusion, as removing morbid heat, and conducing to subsequent diaphoresis, is, in reality, as much an evacuant as purgation. Venesection, however, is the paramount measure, inasmuch as it exercises a mastery over the operation of almost every other evacuant. This was long ago observed by the sagacious Grant, who seems to have anticipated many of the modern correct notions of healthy and unhealthy function.

"At the same time the secretions and excretions become irregular; some are too much promoted, while others are retarded or stopped. Thus there is a degree of heat which promotes insensible perspiration to the degree of sweating, and may be called the sweating point, under which a sweat cannot be produced; but what is more surprising, if the heat is pushed far beyond the sweating point, the skin will become harsh and dry, and we never can recall even the natural perspiration, till the heat is reduced below the point that first produced the sweat. The same is true of every gland in the body. In the beginning of all fevers, there is some degree of spasm, which tempted Hoffman to define a fever, *spasmus universalis.* A seasonable bleeding acts as an anti-spasmodic in many such cases. I have seen in some fevers a vomiting and purging come on spontaneously immediately after bleeding, that has cleared the whole *primæ viæ* critically. I therefore, from reason and constant experience, recommend this observation of Sydenham, that even during the putrid diathesis, when much evacuation is required, in people full of blood, let more or less blood be taken according to the strength and circumstance of the patient, in the first place, and then proceed to other evacuations. Now, if this is proper in the season of the putrid diathesis, surely it must be absolutely necessary during the inflammatory one." *See Grant's Observations on Fevers, &c. P.* 186, 7, 8, *foot note.*

In the foregoing passage, published in 1773, will be found most of the fundamental points so extended by Currie, Hamilton, Armstrong, and the various writers on tropical fever, and which have so lately distinguished the practice of medicine.

In typhus fever, especially of winter and spring, where the respiratory organs laboured under congestion or inflammation (indicated by local pain, scanty respiration, &c.) blood-letting was freely used in any stage, though the safest period, of course, was, at the commencement of the disease. Mucous expectoration, however, having commenced, the lancet was wisely used with caution. Local bleeding, with blisters, may be often ventured on, when general blood-letting would be dangerous. In typhus cases, when the *brain* was the organ principally affected, Dr. P. found the attending debility much greater than in other cases, and the benefit resulting from general venesection much more precarious. He confined himself usually to leeching—arteriotomy in urgent cases—but principally to sponging the shaven head with cold vinegar, or moistening it with æther, while warm fomentations were applied to the lower extremities. After free catharsis or other depletion, blisters to the nucha and between the scapulæ.

In the typhous epidemics of summer, purgatives and cold affusion nearly supersede the lancet. D. P. recommends the afternoon or evening as the best period for bleeding in typhus fever, and justly observes, that "the morning remissions do not furnish a safe guide for the practice of the evening."

The purgatives in general use were combinations of calomel, aloes, scammony, and ipecacuan, with salts and senna. In dangerous tympanitic distention of the abdomen, Dr. P. employed the rectified oil of turpentine with considerable benefit, given in doses of from half a drachm to a drachm.

"The obvious uses of purgatives are to reduce the mass of circulating fluids; to unload the secretory organs, and discharge the vitiated contents of the alimentary canal. *But besides these, I am persuaded that important service is rendered by stimulating the mucous membrane of the viscera, and thus diverting congestion of the peritoneum, pleura, and pia mater.*"

Dr. P. might have added, by rousing the torpid circulation in the portal circle, and giving a freer channel for the blood from the descending aorta.

"It may be remarked, (says Dr. P.) that the old and popular custom in England of taking purges in the spring and fall of the year, to prevent fevers, serves to confirm the value of this practice."

A similar observation is made by Dr. Johnson, at page 157 of his "*Influence of the Atmosphere.*" Of antimonials, Dr. P. speaks unfavourably, as they never proved of much use except as evacuants from the stomach and bowels.

"In general I have found that bleeding, purging, and ablution render them altogether unnecessary in the acute stages. But in those varieties of fever which are protracted by imperfect crisis, where the tongue remains coated, and the skin, though temperate, is dry and torpid, I have employed antimonial powder, in small doses, to great advantage. I have usually combined with it calomel or opium, or both together, and found them highly serviceable in improving the condition of the secretory organs, and amending the discharges of the liver, the mucous membrane, and the skin. *In the dysenteric fever, these remedies are an invaluable resource.* P. 342.

"Of the use of *mineral acids* in typhus fever, I have had little experience, from the incompatibility of administering them freely in conjunction with calomel, *on which much of my reliance is placed in almost all the worst forms of typhus.*" P. 343.

This is strong evidence in favour of the mercurial treatment; but although we have no confidence in the mineral acids, we have reason to know that there is little or nothing to be dreaded from their exhibition with calomel.

The administration of cinchona in typhus fevers was almost entirely confined to convalescence, and then in the form of decoction, with the acidum sulphuricum dilutum or tartrite of antimony.

Of all *cordials,* wine appeared the most salutary; but it was given under careful limitation.

"In all circumstances which indicated cold affusion, viz. morbid heat, a dry skin, loaded bowels, head-ache, &c. wine was pernicious." P. 345.

We need not say that this rule almost proscribes it from general use. If the pulse become more frequent and fluttering under the administration of wine, the remedy is unseasonable; if, on the contrary, the pulse become more regular, soft, and slow, within a certain limit, the salutary operation of the cordial may be presumed.

Opium was not much used in the Hardwicke Hospital. When the bowels were confined, the temperature high, and the skin parched, its effects were injurious; and when the bowels and skin are solvent, and the body cool—rest, sleep, and diaphoresis so commonly succeed, that narcotics are superfluous. Yet there is a variety of typhus in which pervigilium and jactitation are leading features from the commencement to the very crisis. "In all such cases, opium is an invaluable resource." P. 349.—After the first week of fever has elapsed, and evacuations have been duly premised, 15 or 20 drops of laudanum in an effervescing draught or camphor mixture may be given at bed-time.

The treatment of *synochus* may be readily inferred from what has been said of typhus. In respect to the *post mortem* appearances here, our author observes, "I have examined many cases, in which no deviation [in the brain] occurred from the due or ordinary condition of that viscus." P. 355.—The abdominal viscera were the principal theatres of lesion.

There is, in the same volume, an interesting report from John O'Brien, M. D. relative to the fevers in the Cork-Street Hospital. The results of this Gentleman's opinions and practices are so nearly similar to those of Dr. Percival, that we do not deem it necessary to repeat them here. There is one ingenious suggestion, however, which we shall notice.—Dr. Grattan, in the same volume, is not a convert to the doctrine of fever being a *local* disease. On the contrary, he considers it as sometimes accompanied by topical inflammation, and sometimes not. To the former species, he would apply the term *typhitis*; to the latter, the common term typhus. If we could always lay down the limits between inflammation and congestion, and between this last and the general derangement in the balance of the circulation, which most indubitably takes place in *all* fevers, then the distinction of Dr. O'Brien would be practically useful. But we fear that we have not yet arrived at such precise knowledge in the pathology of fever.

Upon the whole, we consider these clinical Reports as assimilating very closely to the doctrines and practices of Dr. Armstrong in this country, allowing for difference of climate and constitution. Epidemic fevers in the metropolis of Ireland will not bear the same system of decisive evacuations as sporadic or even epidemic fevers in this country. The food of the Irish is of a worse quality; their habits are more intemperate in spirituous liquors; their nervous systems are more shattered by a host of depressing passions; by poverty, discontent, and political and religious distractions. Their diseases are thereby modified, and require a more discriminated treatment. But from what we read in these Reports, it is evident that our Irish brethren are not behind us either in theory or practice, in this important class of human afflictions. We anticipate many useful and scientific accessions of knowledge to the common stock from the association now formed in the Irish metropolis.

### III.

*A Treatise on Blood-letting in Fevers.* By J. Van Rotterdam, &c. of Ghent. Translated from the French by J. Taylor, M. D. Octavo, pp. 265. London, 1818.

A perusal of the title page and preface of this work produced an association of ideas not very favourable to the performance. We had twice visited the scene of Mynheer Van Rotterdam's lucubrations, and twice we returned to England disgusted with the van *swamp* doctrines and practices. We venture to say, then, that Dr. Taylor has evinced much indiscrimination in selecting Van Rotterdam's work for translation; and what is worse, that he has undertaken to advocate a bad cause—the doctrine of debility, putrescency; and, to crown the whole, the *medicine expectante* in acute diseases!

It is true, that our author has exercised his wit (perhaps at the expence of his judgment) in drawing a few *caricatures* of the British energetic practice, and holding them up in contrast with the caution and timidity of the van *swamp* measures. "We may also (says he) have reason to remember the effects, when calculated, of *throwing in* bark and wine—of *pouring in* mercury—of *following up* purgatives—of *leeching by relays of dozens*—of *strangling diseases* in their birth," &c. This is mighty witty; and yet Dr. Taylor must give the medical world a very different sample of his practice and opinions, before (to use his own words), we "yield to the influence of *monoculus* principles," and prefer them to the ridiculed precepts of a Hamilton, a Beddoes, and an Armstrong.

Every remedy that has ever yet been useful in disease, has also been *abused,* in the hands of ignorance; but we believe that there is just as much talent and discrimination to be found among the advocates for depletion in fevers generally, as in the ranks of Brunonianism or the Medicina Expectans. Had Dr. Taylor sailed on the four winds, he could not have alighted on a worse school for the treatment of acute diseases, as they appear *in this country,* than on the school of Ghent. We had an opportunity, on the fatal expedition to Walcheren, to contrast the Flemish and British practice in fevers; and in the very year when this Treatise was written, we were again in the vicinity of Ghent, with both the means and the inclination of ascertaining the sentiments of the Flemish practitioners on this point. The result was a conviction that we had little to learn from that quarter. To shew on what foundation Mynheer Van Rotterdam (and we suppose his Translator, who praises him so much) erects his anti-venesectionary doctrines in fever, we shall quote the following passage:—

"It is known that *contagious* diseases are propagated much *less* in cold than in warm climates; although, in the former, the phlogistic diathesis is most frequently met with. What we have said of warm climates applies itself to damp countries, and it is *this* which induces us to use the lancet very cautiously in the swamps of Belgium." P. 13.

Now it is diametrically contrary to fact, that *contagious* diseases are much *less* propagated in cold than in warm climates. The very reverse is the case; and hence the whole hypothesis is founded, *in limine*, on error!

Dr. Van Rotterdam's Translator seems to think that his author deserves great praise for observing what every tyro in the profession knows, or ought to know; namely, that in letting blood in fevers, we must be guided by the season, the climate, the constitution, the age, sex, stage of the disease, nature of the epidemic, &c. Who knows not these things? Dr. V. R. in fact, has strung together a heterogeneous mass of quotations from various authors, and that with not a very fine thread. His work may be calculated for the meridian of Zealand, but it will make a very poor figure among the writings of Hamilton, Currie, Philip, and Armstrong, in this country—leaving aside the many able works on miasmal fevers from the pens of army and navy practitioners.

We cannot refrain from again expressing our regret and surprise that an army surgeon should have attempted, at this time of day, to introduce the pusillanimity of foreign practice in fevers into this country, after what has been written and proved on the subject by his own countrymen. We do not, however, consider the work as of that description that requires a formal examination or refutation. It is most plentifully embued with a certain *plumbean* principle, which will doubtless ensure it a wonderful " alacrity in sinking."

---

### IV.

### *Remarkable Species of* Congestive *Fever, observed by* Dr. Archibald Robertson, R. N. *in the West Indies; with the Appearances on Dissection.*

[Johnson's " *Influence of Tropical Climates,*" second Edition.]

The disease comes on in the usual manner; but from the very beginning, and throughout the whole progress, the nervous system is the chief seat of attack, and appears to suffer dreadfully. Its susceptibility seems to be greatly increased, while, at the same time, its energy is as greatly diminished; hence it is excited into a degree of diseased mobility by impalpable or incognizable stimuli, which are usually inadequate to produce impressions, and loses in a great measure, its healthy influence or controul over the motions, functions, and sensations of particular parts. The patient looks half intoxicated; he staggers; he pants for breath; his voice is shrill and tremulous, and his whole appearance betokens agitation inutterable, with a dash of wildness gleaming at intervals over his anguished features, which add unspeakably to the horrors of this death-bed spectacle. He complains of little or no head-ache, but appears impatient and irritable, yet oppressed; and, after the first day, sinks down in bed with a hopeless or careless expression of resignation. The external senses seem torpid, or are occupied exclusively with ideal perceptions; insomuch that he neither sees nor hears what is going on around him, without a special effort to look or listen; and even then, he is not unfrequently subject to visual or accoustic illusions.

The empire of the will seems to be overthrown, for there is a singular prostration of the voluntary powers, and such tremors of the extremities that the patient is unable to lift a cup of liquid to his mouth; and when raised out of bed, cannot stand erect even for a moment. Under such circumstances, if the cold affusion is administered, the shock induced by it is too much for the morbid nervous susceptibility, and increases the tremors to so fearful a degree, that I have more than once been terrified lest they should end in general convulsions, and have been glad hastily to desist from its use, and to substitute sponging with vinegar and water in its stead.

The organic or involuntary functions are not less disturbed. The stomach is *from the very first*, highly irritable, and so continues. There is also a more than usual burthen about the præcordia, and such a catching in the respiration as caused me to notice dyspnœa as one of the *earliest*, as well as one of the worst symptoms. The heat of the surface is very great, with partial sweats here and there: the pulse, too, is very frequent (from 130 to 140), but easily compressible, and moreover undulating. I do not know a more accurate term than this last to denote the nature of the arterial stroke; for it exactly seems as if each unda of blood followed *instantly* on the preceding one, without an intermediate efficient systole or diastole of the heart. When the patient is raised in bed, or when even an inconsiderable quantity of blood is drawn, (in the recumbent posture) the pulse becomes fluttering and innumerable, and vomiting, with syncope, supervenes. Such is the picture of the patient's state from the first hours of indisposition till the third day of the disease; towards the end of that day subsultus, coma, and cold extremities occur, and death takes place early on the fourth.

"Whenever I have met with this variety, it has proved fatal:—this will scarcely be wondered at, when we view the mixed inflammatory and congestive character of the disease; and when we recollect that blood-letting and cold affusion (our two most potent remedies), are inapplicable in it,—or (which is saying the same thing) can only be very partially and inadequately employed. The peculiarity of this variety consists in the absence of headache, and the pressure of dyspnœa and gastric irritability, from the earliest to the latest hour. The vast commotion of the nervous system, too, and unusual state of the pulse, strongly arrested my attention. I was, therefore, very anxious to see if dissection would throw any light on the symptoms.

"On examining the stomach, I found the vessels on its inner coat much more conspicuous than natural, and filled with dark grumous blood; but without any distinct traces of acute inflammation. In the lungs and other viscera of the thorax and abdomen, there were no appearances of inflammation whatever, and none of congestion, save such as might readily be accounted for by venous gravitation.

"In the brain or its membranes I found few traces of undue vascular actions, save in its basilar portion. *Here* the pia mater was very red, and adhered pretty firmly to the substance of the brain; and patches of coagulable lymph were thrown out at various points, gluing together the inferior convolutions. The tractus optici were loaded with dark-coloured, and apparently infarcted blood-vessels, as were the other cerebral nerves, from the points where they emerge from the substance of the cerebrum, pons, and medulla oblongata, to their exit by the cranial foramina. A tablespoonful of fluid was found in each of the lateral ventricles.

"But it was in the cerebellum and medulla oblongata that the chief morbid appearances were detected; the former shewed a degree of vascularity indicative of high previous inflammation,—anteriorly and inferiorly it was covered with a plexus of distended vessels, and the latter, together with the pons varioli and medulla oblongata, was enveloped by tenacious thready layers of fibrin, which could scarcely be removed without injury to the medullary substance underneath. About two tea-spoonsfuls of serum also were found effused in the fourth ventricle; and the whole of the nerves arising from the medulla oblongata, but especially the par vagum, were found of a purplish colour, evidently from the ramification of minute congested vessels. All the veins and sinuses of the encephalon were greatly distended with blood.*

* I am very sorry that at the time when these cases occurred, I was unacquainted with the interesting researches of Dr. Sanders of Edin-

" Having examined anatomically only two cases, it may appear hasty to attempt any thing like an induction from them; yet, wishing to draw the attention of future observers to this matter, I am inclined to hazard an opinion—speculative certainly, yet not altogether improbable,—that the type and the relative danger of this fever are regulated by that portion of the centre of the nervous system which shall happen to be the chief seat of inflammatory determination and sanguineous congestion. Conformably to this view it might with some shew of reason, be inferred that the danger is less when the anterior and superior lobes are chiefly affected (though the disturbance of the sensorium may be greater); but that the danger is imminent, and death scarcely avoidable when the base of the brain, the origin of the nerves of automatic life, and the medulla oblongata, are implicated in inflammation." P. 452.

How far the appearances on dissection account for the preceding symptoms, is a question which cannot be satisfactorily answered in the present state of our knowledge of pathological physiology; but perhaps it would not be refining too much to suggest, that the morbid vascularity of the eighth pair of nerves (which, with the great sympathetic, supply the lungs, heart, and stomach),—the inflammation of the medulla oblongata, and the compression, too, of this important part from the effused serum, caused that imperfect operation of volition on the parts subject to the will,—that early and permanent debility of the vital functions; that dyspnœa, fluttering pulse and vomiting, which characterised the cases from the first moment of attack.* If I mistake not, Galen has some-

---

burgh, into the state of the spinal marrow and theca vertebralis in general fever; and with the views which he so ably expounds. I now regret in vain the opportunities that then escaped me of investigating this important pathological point.

* " These speculations derive a singular degree of countenance from the results (since detailed to me by my friend *Mr. Hastings,* one of the Presidents of the Royal Medical Society of Edinburgh), which have been observed to follow the dividing of the eighth pair of nerves, in the lower animals. This gentleman,—no less distinguished for his talents than for his zealous and unwearied prosecution of physiological experiment,—has found that, on cutting the par vagum, the process of digestion is completely suspended, and such irritability of stomach induced, that the animal rejects by vomiting all sorts of food or drink. The œsophagus soon becomes paralysed, and deglutition is thenceforth impracticable.

" The action of the heart, also, is greatly weakened and disturbed; and there is a remarkable dyspnœa, which soon increases to laborious breathing, and is followed by the effusion of bloody frothy serum into the air-cells of the lungs, before death. There are also observed reddish patches on the surface of the lungs." P. 452.

where asserted, that inflammation of the cerebellum is always accompanied with an undulating pulse: it was, I believe, this hint, casually stumbled upon, that first led me to examine this and the contiguous portion of the brain with more than ordinary care.

Whatever may be thought of the above opinions, I am satisfied of the accuracy of the fact, that early dyspnœa, and an undulating pulse, viewed merely as pathognomonic signs, always indicate extreme danger in this disease; indeed, when the more ordinary forms of the complaint prove fatal, these symptoms always precede the unhappy event.

---

### V.

*Observations on Dr. Wilson's Tincture, the Eau Medicinale, and other pretended Specifics for Gout, &c.* By William Henry Williams, M.D. of Ipswich. Quarto, pp. 25. London, 1818.

Whoever vends, directly or indirectly, a *secret* remedy for any disease, is a quack. Whether the latent impulse be from WANT, or the object be for wealth, it is all the same: the advertisement of a nostrum is a palpable charlatannerie, which degrades any member of the profession, who, from that instant, *loses cast,* whatever may have been his former rank. From the moment that the Sicilian Tyrant took up the ferula, he forfeited all pretensions to the sceptre. He was as much a schoolmaster as though he had never been a king.

Dr. Williams has, in this work, exposed with great force and acuteness, the prevarications, deceptions, and impositions, which are daily foisted on the public, with the view of picking the sick man's pocket, no matter if at the expence of his life or health! When will government put an end to such diabolical proceedings? The suppression of quackery would be a greater piece of humanity than the abolition of the slave trade; and if some of our sensitive patriots, who shed so many crocodile tears over the *liberty of a libel,* were to interest themselves in behalf of the *lives of their fellow subjects,* they would be more likely to secure the gratitude of future generations.

We shall not follow Dr. Williams through his exposition of that tissue of absurdity and falsehood which interweaves itself with the promulgation of the arthritico-rheumatic specific.

"Dr. Wilson asserts, that 'the three popular medicines for gout, are the colchicum autumnale, the eau medicinale, and his own tincture; but popularity, says Dr. Williams, is a poor and delusive test of merit, if we found its claims on the 'pernicious and fatal effects,' which Dr. Wilson himself ascribes to the two former preparations. Of the third, namely, the tincture, I may declare, with equal truth, from my own observations on its effects, that, it *sometimes proves inefficacious, often violent, and occasionally fatal;* instances of which I shall adduce in the following pages." P. 6.

As among the members of the profession, where alone the pages of this Journal are likely to circulate, there cannot be two opinions relative to the nature of the *charlatanism* under consideration, and the deleterious qualities of the nostrum puffed off, we shall not pursue the subject any further here. We sincerely hope that Dr. Williams's publication, which does equal honour to his head and heart, may be disseminated far and wide among the public at large, that—

The bane and antidote be both before them.

P. S. Since the first edition of this number of the Journal, many instances have occurred where the injurious effects of these nostrums, in gout, have been made abundantly conspicuous. Indeed, we cannot too often warn our professional brethren against the danger of treating gout in any other way than on general principles; always bearing in mind, that the disease is, upon the whole, a salutary effort of the constitution, to keep off an irritation or inflammation from more vital organs or parts.

### Surgery.

### VI.

*On Exostosis.* By Astley Cooper, Esq. F. R. S. &c.

[ Surgical Essays, Part I. ]

It is with no common sensations of pride and pleasure that we open the Surgical Department of our Bibliology with the revered name of Astley Cooper. It is said, indeed, that in the republic of *science*, all feelings of nationality should be entirely suppressed, as beneath the dignity of the philosopher. We class ourselves, however, among those weak-minded, but *warm-blooded* animals, whose hearts swell with exultation, as much at the name of a Newton, as a Nelson; nor do we think that *national pride* is a less effective impulse towards national emulation, and consequently towards national superiority, in science, than in politics. That Briton, who, in future ages, shall navigate the waters of the Nile, or wander over the plains of Waterloo, without *proudly* remembering the names of Nelson and Wellington, will ill deserve to share in the national respect which those heroes have purchased for Englishmen with their blood and valour. And we would ask the question, Can any surgeon of this country see the carotid, subclavian, or iliac arteries tied, in the living subject, and not feel proud, that it was upon British ground such daring efforts to preserve human life were first made? When in future ages, as we confidently trust will be the case, these operations are extended successfully to the aorta itself, will any Englishman be ashamed or afraid to claim for his countryman the honour of having first demonstrated the practicability of such a formidable attempt?

When such men as Cooper, who is deservedly placed in the very first rank of British surgeons, dedicate their few leisure hours to the advancement of surgical science, by communicating the rich store of practical facts which they possess, to their professional brethren, it is an auspicious omen. There is no possible way, in which the healing art can receive such valuable accessions, as by *the evening records of daily experience.* These are not the reveries of fancy, or the transfigurations of the library. "By their fruit ye shall know them," as they bear intrinsic marks of genuine authenticity, which none can fail to recognise. But to our analytical duties.

There are few diseases where the importance of the precept—

Principiis obsta—*venienti* occurrite morbo, is so well exemplified, as in Exostosis. Insidious in its approach, slow in its march, but dreadful in its consequences, it cannot be too early detected by the surgeon, because it is in its nacent movements only that his art may check the disease.

Exostosis, or preternatural growth of bone, may be seated either between the internal surface of the periosteum, and the external surface of the bone; or it may originate in the medullary membrane and cancellated structure of the bone. The former may be termed *periosteal,* the latter *medullary* exostosis. In regard to its nature, it is of two kinds, cartilaginous or fungous. In the first case, there is a preceding formation of cartilage, as a nidus for the ossific deposit; by the *fungous* is to be understood a tumour of softer structure than cartilage, but firmer than fungus in other parts of the body, containing spicula of bone, malignant in its nature, and depending on a peculiar state of constitution and action of vessels; in short, a modification of what Mr. Hey has denominated *fungus hæmatodes.* There is scarcely a bone in the human fabric which may not become the seat of this disease.

1. *Symptomatology of fungous or medullary exostosis.*—This affection evinces itself at first by a general enlargement of the limb, in the part opposite to the seat of the complaint, and to a considerable extent around it. It usually, but not invariably, occurs in young subjects. It increases very gradually, and unaccompanied by much pain; even when it has attained to a considerable size. When pain occurs, it is of an obtuse kind, extending in the course of the bone; but becomes very acute whenever a nerve happens to be brought on the stretch by it, as in exostosis of the femur pressing on the sciatic nerve. The general health is defective. There is paleness, debility, irregularity of the bowels, in the early stages, with a sallowness of complexion, when the disease is confirmed. The limb at length becomes of an enormous size at the disordered part, but the skin retains its natural colour. The tumour feels hard in some parts, elastic in others, conveying to the finger the idea of a subjacent fluid. If an opening be made, nothing issues but blood. The surface of the tumour next becomes tuberculated, and these tubercles are tender to the touch, and frequently slightly inflamed on their surface. Constitutional irritation now supervenes. The rest is broken, the appetite impaired, the bowels deranged. Many weeks pass with these symptoms, and at length ulceration takes place on the tubercles. The skin secretes pus; but when the swelling itself is exposed, it discharges a blood-coloured serum. A fungus then arises, which occasionally bleeds, sometimes profusely, the blood being loose in its coagula, and separating a large proportion of serum.

The bleeding gives a temporary, and only a temporary, relief. The fungus now projects considerably—the skin yields extensively—sloughs—portions of the swelling protrude and come away, and the disease becomes so far diminished in volume, as to induce a hope of its being entirely destroyed by gangrene; a hope which Mr. C. has never seen realized. A great discharge of serum and sanguineous pus now takes place, which, with the constitutional irritation, at length wears out the powers of the body; sometimes in two, sometimes in ten years. In the progress of the disease, and also in cases of amputation, tumours of a similar nature sometimes rise in other parts of the body—even in organs the most essential to life.

2. *Pathology.* The disease originates from the medullary membrane of the bone within the cancelli. The muscles are removed to the distance of three inches or more from the surface of the bone, and form a thin layer over the tumour. The large blood-vessels are next observed to be carried, as well as the muscles, to the vicinity of the surface of the limb. Under the muscles appears the periosteum, separated to different distances from the bone—sometimes two or three inches. The tumour itself is found composed of lobulated masses of various colours, consistence, and materials, some like fat, some like brain, some like coagulated blood, with interstices containing serum. In some places the white substance is found firm, like cartilage, but more spongy, and containing spicula of bone. The shell of the bone itself is, in some places, absorbed; in others, only thinner than usual. In some cases, it is found immensely expanded, so as to form a case like wire-work over the tumour. The fungous granulations themselves are exceedingly vascular, soft in texture, and so luxuriant as to rise from the cavity of the bone considerably above the level of the skin.

An exquisitely marked case of this disease has been related by Dr. Johnson, in the 3rd vol. of the *Med. Chir. Journal,* page 376, where the tumour was taken for an aneurism, and laid open in order to tie the artery. The limb was amputated by Dr. Vance, and the young lady appeared to do well; but about twelve months afterwards she exhibited various and mysterious symptoms of disease in the chest, with a tumour on the left side, which was once more taken for an aneurism of the aorta. After death, however, Dr. Johnson examined the body, and found a tumour, similar to the original one on the thigh, seated partly within and partly without the thorax, like a double headed shot; the middle part being situated between two ribs, to both of which it adhered. Another tumour, of a similar nature, rose from the spine, and actually forced the heart from its natural site. Hydro-pericardium was, of course, the consequence.

3. *Etiology.* Nothing certain is known respecting the cause of fungous medullary exostosis. In some instances it has been attributed to a blow; in others, to a jump from a height. Either of these causes, by disturbing the interior action of the bone, might produce the effect. The fungous species of the disease generally, however, depends on a faulty state of the constitution.

4. *Treatment.* This is both local and general. It is needless, however, to observe that when any considerable change of structure has once taken place, with much increase of parts, no medical means will suffice to restore them, or prevent the fatal tendency of the complaint. "But in the commencement of any deep-seated disease in bone, the best medicine, so far as I have had an opportunity of observing, is the oxymurias hydrargyri in small doses, given either in, or with the decot. sarsæ compositum. This mercurial medicine, by reproducing the natural secretions of the body, and the sarsaparilla, by lessening its irritability, restore the general health, and will sometimes crush in its bud a disease, otherwise likely to become formidable, and at the same time prevent the formation of a similar affection in other structures." 169.

The local treatment consists in the application of leeches, if there be pain, and of blisters, taking care to keep up the discharge from their surfaces by means of savin and mercurial ointments, of each equal parts. Even should the disease not yield to these, the patient will be rendered a better subject for an operation for the removal of the parts, which then becomes the only resource. Mr. C. tried the effect of cutting off the supply of blood, by tying the artery which leads to the tumour, but without success.

5. *Cartilaginous Exostosis of the medullary membrane.* This differs greatly, both in appearance and nature, from the above described disease. Here the shell of the bone becomes extremely expanded; or rather the original shell is absorbed, and a new one deposited, within the cavity of which, a large mass of cartilage is formed, elastic, firm, and fibrous.

In its commencement, there is no malignity. It arises from common inflammation in a healthy constitution; but the long continued irritation brings on extensive disease.

6. *Treatment.* When the *cause* can be traced satisfactorily, as for instance to the irritation of a decayed tooth in the jaw-bone, its removal ought first to be attended to. Should this prove insufficient, it will be necessary to take off the external shell of the bone by means of a saw, and then dislodge the subjacent cartilage by an elevation. If the integuments be carefully preserved, little deformity follows; and thus, by a simple operation, destruction, otherwise inevitable, is prevented.

7. *Periosteal Exostosis.* This disease, like the preceding, may be either *fungous* or *cartilaginous.* The *former* scarcely differs in its symptoms from the fungous exostosis of the medullary membrane, except that the general swelling of the limb is less, and the tumour itself is more prominent; but there is the same want of sensibility in the commencement, with succeeding pain. The skin remains free from discolouration, and has a similar tuberculated appearance. Ulceration, hæmorrhage, sloughing, and general discharge ensue, and occasion the destruction of life, if some operation be not performed. The following case will illustrate the symptomatology and pathology of this disease.

*Case.* "A girl, 19 years of age, was admitted into Guy's Hospital for what was at first supposed to be an enlargement of the knee joint, but upon more particular examination, it was discovered that the swelling occupied the lower part of the os femoris, to which it was immoveably attached. The countenance of this girl was sallow, her general health appearing extremely defective. The swelling was small at her first admission, but during the time she was in the hospital, it rapidly increased; the skin was undiscoloured, and the surface of the tumour was tuberculated, hard as bone in some parts, but elastic in others. It was at first entirely unattended with pain; but as it increased it became occasionally extremely painful, and evidently reacted upon her constitution in such a manner as to threaten her life, unless the operation of amputation should be had recourse to. The limb was consequently removed. Violent constitutional irritation succeeded to the operation, which for several days excited an apprehension for her life.

"When these symptoms subsided, the stump put on an unhealthy appearance. Its irritability was excessive, so that she dreaded extremely the approach of her medical attendants for the purpose of changing the local applications. A fungus arose from the cancellated structure of the bone, which it was necessary to destroy with caustic. Many weeks elapsed before the closing of the stump, notwithstanding a sufficient quantity of integuments had been preserved. And, indeed, at length, when she was discharged from the hospital, some slight ulceration of its surface was still remaining: but it was thought adviseable that she should have the advantage of a more salubrious air than that of an hospital in a large town.

"DISSECTION. The exostosis was seated at the lower part of the femur; the periosteum passed over it, and adhered strongly to its surface. The tumour itself was very firmly fixed to the external surface of the shell of the bone. It was injected minutely with size. In some parts it appeared extremely red with the injection; in others, where the injection would not enter, it was white, so that it was found very vascular in some parts, and in others not at all so. The surface of the tumour was lobulated. The periosteum at one part appeared to have formed, upon its external surface, a tumour composed of similar materials to that which was seated between it and the bone." 182.

8. *Etiology*. The disease is often attributed to accident; but any irritation upon a bone in an unhealthy constitution will produce it. A fine specimen is preserved in Guy's Hospital, arising from an internal exfoliation of the os femoris.

9. *Treatment*. This is similar to that required in the fungous exostosis from the medullary membrane; (iv.) but it is only in the first dawn of the disease that we are to entertain any hopes of a benign influence from medicine. "It would be dishonest (says Mr. Cooper) to assert that we have a knowledge at present, of any medicine having a specific influence over cancer or fungus. We may, indeed, improve the constitution a little, and keep the disease at bay; but once formed, it proceeds more or less rapidly to its fatal termination, unless prevented by the operation of amputation or excision; which the state of the constitution, improved by medicine, will render more safe at the moment, and hold out a better ground of hope for the future."

10. *Cartilaginous Exostosis, between the periosteum and the bone.* This differs greatly from the preceding, and is more deserving the attention of the Surgeon, since it admits of relief by operation, though sometimes with the loss of the affected limb. It originates in inflammation of the periosteum and of the corresponding part of the bone; while a deposition of cartilage, of very firm texture, and similar to that which forms the nidus of bone in the young subject, adheres to both these surfaces. The periosteum adheres to the external surface of the swelling, and the swelling itself is attached still more strongly to the surface of the bone. Within this cartilage a bony matter is deposited, which is first thrown out from the original bone. It continues afterwards to be secreted as the cartilage increases in bulk; for it appears that between the periosteum and bony mass, cartilage is constantly secreted, which constitutes the exterior surface of this tumour.

11. *Appearances on Dissection.* Here we discover, 1st, the periosteum thicker than natural. 2nd, the cartilage immediately below the periosteum. 3rd, ossific matter deposited within the cartilage, and extending from the shell of the bone nearly to the internal surface of the periosteum, still leaving on the surface of the swelling a thin portion of cartilage unossified.

12. *Symptomatology.* For the most part these diseases are attended with very little pain, especially at their commencement. But when they have acquired some considerable size, they occasion painful sensations by their pressure upon surrounding parts. Very great inconvenience also arises to the patients, from the impediment to the action of the muscles, the tendons of which are sometimes detained in particular positions; while at others, they glide suddenly over the tumours with a snapping noise, which can be distinctly heard, and accompanied by very painful sensations. The most frequent seat of the disease is upon the inner side of the os femoris, just above the internal condyle, and in the direction of the insertion of the triceps muscle. Mr. C. has also seen it seated on the tibia, under the insertion of the Sartorius and Gracilis muscles. It sometimes affects the fibula at its connection with the tibia. After long continued courses of mercury, when the patient has been debilitated to an extreme degree, if he exert himself much in walking, not only is this thickening of the bone of the fibula produced, but a suppurative process is instituted, which is followed by exfoliation, and lays the foundation of a tedious, and sometimes dangerous disease.

13. *Etiology of Periosteal Exostosis.*—Where this has a small base, and follows the course of the tendons or ligaments, Mr. C. thinks it owing to exertions disproportioned to the strength of the subject. The disease denominated *splent* in horses, is produced by sprains of the ligaments, and is exostosis. Blows also occasionally produce the disease.

14. *Treatment of Periosteal Exostosis.*—It admits of relief from internal medicine, from external applications, and when far advanced, from surgical operation. The internal and local treatment differs in no respect from that of medullary exostosis. (iv.)

"'The common alterative plan of small doses of mercury, with decoction of sarsaparilla, combined with stimulating plasters, as the emp. ammon. c. hyd. with the view of promoting absorption of that which has been effused by its stimulating qualities and by its pressure, are the means which are generally adopted. But in this instance, as in the former, my experience does not furnish me with an example of such remedies having any influence, except in the very commencement of the disease; and too often the insensibility of such swellings prevents their being discovered until they have acquired some magnitude." P. 197.

When they have arrived at a considerable size, they sometimes remain stationary, and produce no inconvenience. At other times they continue to grow, and to interfere so much with the motions and functions of surrounding parts as to render their removal necessary.

15. *Operation.*—This is performed by means of a saw, with little pain and no danger, if the disease be accurately discriminated from the fungous species. Mr. Machel's saw is the best for that purpose, as it admits of being applied at a considerable depth amongst the muscles without doing them any injury. Several very interesting cases are related in elucidation. Mr. Cooper concludes thus,—

"It appears then, that bones, after operations, unite by adhesion to the soft parts; if adhesion cannot be produced, healthy granulations arise from the surface of the bone, and cicatrization takes place upon these, as upon other parts of the body; and that there is reason to believe, that these structures may, with properly constructed instruments, become much more the subjects of operations than they have hitherto been considered."

Mr. Cooper, in his writings, as in his practice, goes directly forward to his object with equal confidence, and knowledge of the subject. He wisely disdains to have recourse to those meretricious trappings with which some medical authors ornament their productions; for as "good wine needs no bush," so the statements of a Cooper require not the authority of a Celsus, an Aretæus, or even a John Hunter, to prove their passports to public credence and professional respect.

---

### VII.

### *On Iritis.* By Benjamin Travers, Esq. Surgeon to St. Thomas's Hospital.

[ Surgical Essays, Part I. ]

In the medical as in the political world—

"Some are, and must be, greater than the rest."

Those noble Institutions of the metropolis, some of them devoted to single objects, as the Lock Hospital; Eye Infirmary, &c. offer to men of talent facilities in the pursuit of scientific investigations, which enable them to far out-strip their brethren who are debarred from these advantages. When such men, therefore, come forward, and generously communicate the knowledge thus acquired, they confer an invaluable benefit on society at large, and justly merit the undivided gratitude of the profession. Mr. Cooper and Mr. Travers have shewn an example in this respect, that is highly worthy of imitation by all the superior medical officers of public hospitals. A similar liberality of spirit and professional ardour have sprung up in the metropolis of the sister isle; and the Dublin Hospital Reports, the Transactions of the Irish College Association, the London College Transactions, the Medico-Chirurgical Transactions, and the various periodical journals, in this country, will now pour a flood of knowledge and professional improvement through every department, and among every class of medical society. A greater equilibrium of information between the upper and lower orders of the profession, must inevitably result from this; and the natural consequence will be, that gradual and progressive *reform*, with its accompaniments, dignity and public estimation, which no act of parliament or academic seal can ever confer!

In launching our little bark on this wide ocean of medical literature, in order to

"Share the triumph and partake the gale,"

we trust that we shall not prove either *useless* or negligent navigators. If our vessel be *small* and a *slow* sailor, she will not often be found performing her voyage *in ballast*, for want of a cargo. Her size too, will enable her to wind among shoals and navigate inlets, where larger vessels could not approach. Her pilots, in fine, are resolved to leave no *creek* or corner unexplored, where *useful* materials can be found for the general mart of medical science.

The paper which forms the subject of this Report exhibits a fine specimen of analytical investigation, and displays an accuracy of discrimination, a closeness of observation, and a soundness of judgment, that stamp the seal of merit on every thing from the same pen.

By Iritis Mr. T. means to express a *deep-seated* inflammation of the eye, whether arising as an idiopathic affection, or from extension of a more superficial inflammation. It often alternates with, or accompanies chronic rheumatism and gout; but it is in its connection with, or dependence on syphilis and mercurial action, that our author here considers it. Its occurrence, during the use of mercury, is now a well-established fact, and Mr. T. thinks it a more frequent *consequence* of this medicine, than as an idiopathic disease; at the same time he acknowledges that

"It appears to him, at present, impossible to pronounce whether the iritis, so frequently presented after sores on the genitals, and accompanied by eruptions, is the effect of a morbid poison, or of the mercurial poison; or, thirdly, the casual effect of exposure to an exciting cause in a state of predisposition from the mercurial impregnation of the system." P. 61.

When once a morbid poison, as the syphilitic, has passed into the circulating from the absorbent system, we entirely lose all clue towards the *modus operandi* of its further action on the constitution. The lymph effused upon the iris is as probable an effect of venereal inflammation, as when it is deposited upon the periosteum.

"The sympathy or consent which obtains between certain parts, appears to influence the course of their diseased actions. The throat, skin, and bones, are observed to be affected by the poison of mercury as well as of lues, and also by that which resembles lues, except in its curableness by other means than the use of mercury. It is remarkable that the eye frequently forms a link of this chain; or, in other words, that the inflammation of the choroid and iris co-exists with affections of the throat, skin, and bones; whether these be syphilitic, pseudo-syphilitic, mercurial, or rheumatic. It is also remarkable, that the remedy which exerts the most powerful and obvious effect upon the inflamed periosteum and skin, acts with the same remarkable efficacy upon the inflamed tunics of the eye, and *vice versa.* Depositions beneath the periosteum, resembling nodes, and eruptions on the skin, are not less certainly induced by the poison of mercury, than is the deposition upon the choroid, ending in incurable amaurosis."

Mr. T. justly observes, that the difficulty of deciding whether iritis is determined by the syphilitic or mercurial poison, arises from the complication of the cases; since the former is rarely seen genuine and unsophisticated. This, in truth, is the cause of that obscurity which overhangs the whole subject of venereal disease.

"It is, perhaps, impossible to say to which of two agents the effects belong, which manifest themselves at a period subsequent to their successive introduction. Both, we are taught to believe, cannot be at the same time active; but both may be present, or one only may, in fact, have entered the system. When mercury is exhibited, as it almost constantly is before the constitutional signs of lues appear, what demonstration can be had of the existence of syphilis in a secondary form? Or, on the other hand, who, in his own case, would, *a priori*, abandon the operation of mercury, in confidence that the symptoms which are occasionally referred to its operation, are wholly independent of the poison of syphilis." P. 64.

Few indeed, we think, would risk this trial on their own persons.

That mercury, however, sometimes induces iritis independently of syphilitic virus, Mr. T. is convinced, from seeing the disease follow the exhibition of the medicine for sores on the penis, which healed without producing any secondary symptoms. He has also seen it follow a useless mercurialization for gonorrhœa, and the free exhibition of calomel in scrofulous inflammation of the eye. In the latter case, he has seen repeatedly

"The iris take an inflammatory action, and while the cure of the iris was daily *accomplishing by the action of mercury in one eye*, it was common to observe the inflammation beginning in the other, as if the action produced effects diametrically opposite upon the sound and inflamed part." P. 65.

Mr. Travers states that, with one exception,

"He does not call to mind a well ascertained case of inflamed iris, *during the constitutional action of mercury for a disease* in which the genitals had no concern, as enlargements of the liver, &c." P. 66.

This is surely a proof, amounting almost to demonstration, that the genital defœdation has either a part in the production of iritis, or lays a predisposition to it, which amounts to nearly the same thing. The pains accompanying this peculiar inflammation are of a rheumatic character.

1. *Description of Idiopathic Iritis.*—In a recent stage, there is diffused vascularity of the conjunctiva; increased sensibility of the eye; the fine hair-like vessels of the iris are injected with red blood; the pupil, though not so obedient to the alterations of light, as in the natural state, is not misshapen or differing much from the circular. Sometimes small specks of extravasated blood are perceptible upon this membrane; sense of pain, and pressure in the eye-ball. In a more advanced stage the fibres of the iris are agglutinated; its pupillar edge is thickened and immoveable, a boss or layer of lymph is discerned upon it; the vascularity of the conjunctiva is less; the bright florid colour of the blood vessels being exchanged for one of a deeper and more purple cast. The vessels of the sclerotic run in converging lines from all sides of the visible hemisphere of the globe, and form a remarkable vascular zone at the circumference of the cornea. The pupil is now square, oblong, rhomboidal, or octagonal; and a boss or tubercle of lymph is deposited upon one or each of the angles which are formed in its margin. In this state, the pain is hemicranial, and most intolerable at night.

2. *Chronic form of the disease.*—This differs from the former chiefly in its very gradual access; its comparatively moderate pain, affecting only the ball and region of the orbit; its slight degree of superficial vascularity; the membranous form and sparing quantity of lymph effused, or its actual imperceptibility; the frequent concomitant affection of the cornea, with minute and superficial herpetic ulcers, of a brown colour.

3. *Hydrargyro-syphilitic Iritis.*—This, so far as Mr. T. has observed, is principally characterised by a brick-dust or dusky red, instead of a scarlet hue in the inflamed conjunctiva and sclerotica; while the deposited lymph is compact and brown, and intimately adheres to the iris, instead of being curd-like, loose and of a yellowish white colour.

"When the conjunctiva is highly vascular and florid, as in common ophthalmia, and the lymph is diffused, white, loose in texture, and puriform in appearance, especially where the cornea is at the same time clouded, and the eye very irritable to light, I have considered the case to be essentially unconnected with syphilis. The irritability of the eye to the light has been considered as diagnostic, and it certainly is so, between the primary iritis and that which ensues upon or is accompanied by acute inflammation of the conjunctiva. In the *latter* case the cornea is hazy, and the aqueous humour turbid; yet the vision is not more affected than in the inflamed iris which has proceeded to the deposition of lymph." P. 69.

4. *Treatment.*—"The iritis first described, (idiopathic) which often supervenes upon indecisive or mistaken treatment of inflamed conjunctiva, or upon some imprudent use or exposure of the eye in this state, is cured by large and repeated blood-letting and active purgatives." 78.

Here we cannot help remarking, that we have seen, with sorrow and disappointment, our patients almost *exanguious* from *general* blood-letting, while the local inflammation kept on its pace uninterruptedly. We would, therefore, strongly recommend *local* detractions of blood, in conjunction with those depletory and antiphlogistic measures which experience has sanctioned, rather than enormous abstractions from the general system, which many surgeons are too much in the habit of making.

Mr. T. from the cases detailed, is evidently of this opinion.

"All the other forms of iritis, says Mr. Travers, whether primary or secondary, simple or specific, require the constitutional use of mercury for their cure without exception. This may be boldly stated, without reference to the organ of the disease, and I should be quite at a loss to name any other disease which so certainly, and so rapidly yields to a stated remedy." P. 78.

Now here is the enigma. Mercury, like the spear of Achilles, has the power of healing the wound it inflicts!

"The beneficial use of mercury in iritis, strange to say, is an observation of but a few years' date. Its use was still more recently confined to the cases which were combined with traces of the syphilitic poison. But averse as are European practitioners, from education or prejudice, or both, for they are not always unconnected, *to introduce mercury into the system during a state of active inflammation*, it is now, by a multitude of facts, incontestibly established as a remedy of unfailing efficacy *in the most acute form*, and in every variety of inflammation of the iris. The ascertaining and promulgation of this fact are due to the Infirmary of this Metropolis for Diseases of the Eye; and in the catalogue of modern contributions to medical science, except the practice of Vaccination, I know of none entitled to rank before it." Page 80.

In the active and early stage of acute inflammation, blood-letting and purgatives should be premised. We hope this statement, and from such authority, will have its weight with those who, associating the idea of *stimulant* with mercury, hypothetically proscribe one of the most powerful anti-inflammatory remedies we possess, where there is any pyrexia or inflammation going on in any part of the system. Calomel and opium, in varied doses, was the form used till the mouth became sore, when the inflammation always subsided.

5.—*Modus Operandi of Mercury.*—Under this head, Mr. Travers has made many judicious observations and ingenious reflections. His ideas very nearly coincide with our own on this subject, and we are proud of such a coincidence of opinion. The effects of mercury in iritis, he remarks, are capable of extension and application to other organs in a similar condition. They afford demonstration the most palpable, that mercury has power to alter the action of the extreme vessels which are the instruments of morbid changes in all organs of the body.

"When it has entered the system, the febrile irritability is allayed, and a general tranquillity prevails, *for all the secretions of*

*the body are manifestly increased, the biliary and cutaneous especially.* The absorbent vessels are next reciprocally excited to increased activity; and where its effect is salutary, it is at this time that the patient experiences a relief as from a burthen, a return of appetite and digestive power, and sense of renovation." P. 89.

6. *Dr. Farre's Opinion.* Mr. Travers has published a highly-interesting letter from that excellent pathologist, Dr. Farre, from which we shall quote a few extracts:—

"Whilst the principal tendency of that series of remedies, which we comprise under the received term, Antiphlogistic, from general blood-letting downwards, is to diminish the force of the heart and arteries; it is in a peculiar manner the operation of mercury on the whole arterial capillary system to change its action, but not indefinitely. The gentlest action of mercury is to correct and restore the secretions proper to the alimentary canal to their natural condition, and, as by a charm, *to dissolve the functional disorder of distant organs sympathising with the first passages.*—This is an operation which so exactly accords with the intention of Nature, that no morbid action ought to result from the remedy itself when thus used."

He conceives that the condition of the extreme vessels, when fully excited by mercury, is an erythema, an action which essentially *weakens the cohesion* of parts:—

"But the adhesive inflammation is *so exactly opposed to this*, that both cannot be the result of mercurial action. From the moment that I commenced the study of morbid anatomy, I directed my attention to the *adhesive inflammation*, because it opened to my view the most usual process of disorganization in the viscera."

"Suffice it to say, that I have been led from repeated observation of the adhesive inflammation of various textures, being cured by the mercurial action, to receive it as one of the general laws of its operation to change that arterial action on which the effusion of coagulable lymph depends, and consequently to arrest all the subsequent changes which flow from this process." P. 92.

"I have uniformly regarded the mercurial as one of the most effectual means of arresting the disorganizing process of adhesive inflammation, whether of the iris or of any other texture of the body. To the liver in this state of disease (Hepatitis) it has been long applied, *except that some have had their fears about commencing it too early; and through this delay, have probably lost the opportunity of preventing suppuration.* In cynanche trachealis it has been more recently used with success. In the last stage of marasmus, from nodes of the large bones, I applied it with success in 1805, and since that period, with equal success, to adhesive inflammation of the pericranium, both where it has been entitled pseudo-syphilitic, and where it was neither syphilitic nor bearing any resemblance to syphilis. Before and since that period I have exhibited it with marked advantage in arterial congestion, and even in organic changes of the brain; in 1809 successfully in *carditis from acute rheumatism*, and since that period, in *chronic carditis*." P. 98.

These are important facts, and well worth the attention of the faculty at this moment, when a host of visionaries are exerting themselves to banish mercury from the Materia Medica. We beg leave to remark, however, that we are of opinion that both Mr. Travers and Dr. Farre have overlooked an important feature in the *modus operandi* of mercury; namely, the great power which it possesses of equalizing the balance of the circulation and excitement, at the same time that it restores suspended functions and enlivens various torpid secretions. We beg that these distinguished cultivators of medical science will peruse with attention what an ingenious foreigner has said in the *third department* of this Journal. *See Vialle on Irritation.*

We have now only to subjoin a short case, as a specimen of Mr. Travers's practice in *Iritis.*

"*January* 30, 1817. Eliz. Scarlet, aged 18, had sores on the pudendum, and a bubo in each groin, fourteen months ago, for which she rubbed in mercurial ointment, for 35 successive days. She continued free from complaint until three months since, when she was attacked with pain in the joints, exacerbating at night, and eruptions on various parts of her body. Shortly after the eyes inflamed, with the left of which she has been unable to see for several weeks. The iris of this eye is deeply inflamed, a zone of chocolate-coloured lymph surrounding the pupil; and eruptions of a dingy red colour are sparingly diffused over the face, arms, back, thighs, and legs, commencing in small white vesicles, which break, and are succeeded by reddish brown laminæ of cuticle. Ordered unguentum hydrargyri, six leeches to the eyelids, a collyrium composed of a scruple of superacet. plumbi to six ounces of water. *Feb.* 10*th*, Mouth sore; ophthalmia and eruptions declining. *Feb.* 19*th*, Mouth very sore during the last week; pains much relieved, and the eruptions faded; vision of the affected eye is permanently impaired, but the lymph is taken up, and it is quite free from inflammation. *March* 12*th*, discharged cured."

We need not apologize for the minuteness with which we have analysed this paper of Mr. Travers. The eye is an important organ; and few accidents are more injurious to the professional character of the *private practitioner* than a miscarriage in ophthalmic inflammation, ending in the loss of sight. It is his duty, therefore, as well as his interest, to study with peculiar care every affection to which the delicate visual orb is subject. To Mr. Travers the thanks of his brethren are due, and will not be withheld. May he persevere with energy in the path where he has commenced with such ability!

### VIII.

*On Periostitis.*—By PHILIP CRAMPTON, M. D. Surgeon-General to the Army in Ireland. 27 pages.

[Dublin Hospital Reports, Vol. 1.]

Inflammation of the periosteum frequently occurs, as a local disease, and is very important in its consequences. It has not, however, been treated of separately, as neither Ploucquet nor Young make any allusion to it. The decisive treatment adopted in paronychia periostei (the severest form of deep seated whitlow) has not been extended to painful nodes of the cranium, tibia or sternum, although these last are similar in their nature to the former, are often equally idiopathic, and as certainly relievable by anlogous treatment. It is very generally considered as symptomatic of some specific constitutional disease, or as the consequence of a diseased state of the subjacent bone; but Dr. C's object, in this paper, is to direct the attention of surgeons to it as an idiopathic affection.

It is a severe disease. The intolerable pain, and the constitutional disturbance which it occasions are referable to the peculiar structure and vital properties which the periosteum enjoys in common with the *fibrous system* of Bichat, of which it forms a part. In structure it is inelastic, and incapable of yielding (without rupture) to an extending cause. In respect to vital properties, like tendon, ligament, and aponeurosis, it is insensible, unless in a state of inflammation, when its sensibility is excited to the highest degree. Periostitis, like other inflammations, may be acute or chronic. These states, however, are only the extremes of a scale which the disease traverses in various directions, according to circumstances. The paronychia periostei affords a very pure example of the acute form; an affection which is to be distinguished from the more usual form of paronychia, in which the inflammation is seated in the *sheath* of the flexor tendons of the fingers. In the paronychia periostei the pain is excruciating, with proportional disturbance of constitution. The finger, which is the seat of the disease, and soon afterwards the hand, become swollen and tense almost to bursting. Erysipelatous inflammation, with œdema, quickly extends over the hand and arm; the fingers become livid, and if a timely operation be not performed, the disease terminates in sphacelus, and the destruction of one or more of the phalanges. But this is not all. The mortification sometimes extends to the integuments of the hand, and even of the fore-arm; deep-seated abscesses form among the muscles; and after weeks, or even months of suffering, the patient may be esteemed fortunate if he escape with a stiffened and mutilated hand. All this too from a neglected inflammation of the periosteum of a single phalanx!

In the paronychia of the sheath, the suppuration finds its way to the surface through a small opening, which soon throws up a painful fungus, perforated in the centre, through which a probe may be readily passed down to the tendon, but no farther. In the par. periostei, on the contrary, the disease, if left to nature, uniformly produces sphacelus of the soft parts, and the probe passes down at once to the naked bone. This short digression respecting paronychia may be excused, when we consider the vast importance of a complaint which may maim an individual for life, and thus reduce himself and family to beggary, all which may be prevented by a timely stroke of the scalpel! But to return.

The terminations of periostitis are various, the acute form generally ending in suppuration, the chronic in cartilaginous thickening of the tissue, absorption of the subjacent bone, or deposition of bony matter on its surface.

Dr. C. justly, we think, admits that—

"Periostitis, like other diseases commonly called local, is, in general, *connected* with some constitutional derangement. It accordingly appears most frequently in scrofulous habits, and in those whose constitutions have been broken down by repeated courses of mercury, or by a long residence in hot climates. I have, notwithstanding, met with the disease in persons who, in every other particular, seemed to enjoy the most perfect health." P. 335.

Dr. C. believes external injury to be a more frequent exciting cause of this affection than is generally supposed. He relates three examples of the disease in its acute form, the principal features of which we shall condense.

*Case* 1. A man, 26 years of age, was found by Dr. C. crying out with pain; his face pale, and bedewed with a cold sweat. Upon the upper and flat surface of the tibia there was a diffused tumour of a pale red colour, extending about two inches below the articulation of the knee-joint, smooth, hard, and exquisitely painful to the touch. He had not slept for twelve nights, and could retain nothing on his stomach. Pulse 124, small and weak; tongue white. He had had a similar attack two years before, which ended in the exfoliation of a thin scale of bone. Dr. C. immediately made an incision about three inches long, down to the bone. The operation seemed to give great pain, the wound bled freely, no purulent matter escaped. He passed an excellent night, quite free from pain. The wound was dressed superficially, and healed in a short time. Three years afterward he experienced a similar attack farther down the tibia, and was speedily cured by the same means.

*Case* 2. Master T. ætat. 14, subject to febriculæ, was attacked on the 1st September, 1816, with a small angry tumour on the right side of the bony arch of the nose, which, in a day or two, became erysipelatous. 5th, Feverish symptoms ran high, although he had used fomentations, purgatives, and had lost 12 ounces of blood. 6th, Venesection to 16 ounces, with apparent relief. 8th, The swelling has extended to the right eyelid; pulse hard and strong; repeat the venesection. 9th, In a consultation, the disease appeared so far mitigated that attention to the state of the bowels only was thought necessary: But in the evening he became comatose, with a slow full pulse, and occasional delirium. Venesection to 16 oz. and a blister to the neck. 10th, A violent convulsive fit, after which there was coma with stertorous breathing; pulse 120, full and throbbing; integuments of the nose, right eye-lid, and teguments of the forehead erysipelatous; both eyes closed; exquisite pain on pressure of the parts; some pus from the right nostril. Temporal arteriotomy; brachial venesection to 20 ounces, sixteen leeches to the temples, ice to the head, which afford much temporary mitigation of the pain, but no eventual advantage. *Evening*, 20 ounces of blood from the temporal artery. 11th, Moaned much, but could not articulate; pupils appeared large and immovable, with a layer of coagulable lymph over the cornea of both eyes. Leeches, blister, &c. were resorted to, but he died on the morning of the 12th, without any convulsive struggles.

*Post Mortem Appearances.* Pericranium covering the os frontis of the right side, thicker than natural, and, in some places, of a dark red colour; it was completely detached from the subjacent bone, and its *inner* surface was covered with thick purulent matter, of a light green colour. The detached and purulent state extended backwards into the orbit, and downwards behind the temporal muscle. On raising the skull-cap, the *outer* surface of the dura mater (for an extent corresponding with the limits of the detached pericranium) was separated from the internal table of the skull, presenting a dull flocculent surface, and smeared with a greenish puriform mucus. The structure of the *inner* surface of the dura mater was less altered, but more generally covered with purulent matter. The brain was more vascular than natural, and about an ounce and a half of water in the ventricles. The periosteum of the nasal bones was in a state of suppuration, and so extensively detached from the subjacent parts, that a probe could be passed in all directions between them.

It appears therefore that the original disease was periostitis of the nasal bones, ultimately spreading to the membranes covering the bones of the face, and at last to the investing membrane of the brain itself.

*Case* 3. Mary Loudon, aged 32, admitted into the Meath Hospital 14th December, 1812, complaining of intense pain of the head, chiefly affecting the left side; right arm wasted and paralytic, lower extremities feeble; speech faultering, countenance pale, sleeplessness; pulse 60, small, weak, and compressible; gastric irritability; torpid bowels; had a strong convulsive fit soon after received. A tumour, about the size of a walnut, over the middle of the left parietal bone, soft and elastic in the centre, firm in the circumference. There was a small opening in the apex, through which a probe could be passed down to the bone. *History.* Six months previously, her husband struck her with the heel of his boot, which stunned her, but did not wound the integuments. She continued well for a month or six weeks, when a swelling with considerable soreness of the parts were perceived. Tumour became daily more painful, with head-ache, sickness of the stomach, insomnium, general deterioration of health; and in five months from the receipt of the injury, an epileptic seizure followed by paralysis of the right arm, and difficulty of articulation, &c. These epileptic paroxysms had frequently recurred since, and her health and spirits had rapidly declined.

On the 18th of August the tumour was laid open, in order to trephine the skull. The pericranium, which formed the bulk of the tumour, exhibited a firm fibrous structure; great vascularity and sensibility, with the most tenacious adherence to the subjacent bone. Outer table of the scull scabrous from absorption, but still the pericranium firmly adhering to it. When the circular portion of bone was removed, the dura mater bled freely; the membrane seemed to have undergone a change of structure, in all respects similar to that in the pericranium. Wound dressed simply. Nothing material till the 22d, when, at eight o'clock, a severe rigor took place. She can now with difficulty be roused; breathing laborious, stertor, countenance pale and sunken, sometimes suddenly suffused with red; pulse 120, small, and indistinct; extremities cold, tongue dry, brown in the middle; bowels constipated; stomach rejects every thing; purgatives continued. After several days of imminent danger, the bowels at length yielded to purgatives, and all the alarming phenomena gradually disappeared. On the 1st of September she was free from complaint.

It is pretty evident that in this case the disease originated in the pericranium, and was transmitted from thence to the dura mater. But in which ever membrane it begins, reason and experience suggest the propriety of dividing the inflamed pericranium, whether with the view of preventing the extension of inflammation to the dura mater, or of relieving that membrane, should it already have been inflamed.

*Case* 4. *Subacute form.* Mr. B. gave the following history of his complaint. In the spring of 1812 he became affected with periodical pains in the upper part of the tibia of the right leg, which was tender to the touch, but neither swelled nor discoloured. Leeches, blisters, and pills (he thought mercurial) gave but temporary relief, and his general health declined, with evening fever, and subsequent perspiration. A roundish flat tumour now appeared on the upper part of the tibia, and the leg was œdematous. Mr. Pack, of Kilkenny, told him it was periostitis, and made an incision down to the bone, with the most complete success. The pain was immediately relieved, and in a few weeks Mr. B. was restored to health. Early in March, 1814, the disease recurred, but a little lower down. The evening pains were, as before, terminated by profuse perspiration. His appetite failed him, and he emaciated fast. Mr. B. noticed a curious fact in his own person, illustrative of the influence of fermented liquors on inflammatory diseases. He could, at any time, bring on the paroxysm of pain in a hour after drinking a glass of spirituous or vinous liquors.

He was now put on a course of blue pill and decoctum sarsæ. An incision was made down to the bone; the periosteum was found a quarter of an inch in thickness, its structure being nearly cartilaginous, and its sensibility much increased. The success was complete, though the operation was again performed lower down.

*Chronic Periostitis.* This form may exist for a considerable time without much deranging the general health; but it is not, on that account, the less dangerous. Sooner or later the subjacent bone becomes diseased; and as has been seen, when the pericranium is the seat of the affection, the most serious consequences are to be apprehended from extension of the disease to the dura mater.

Dr. C. considers chronic inflammation of the pericranium as intimately connected with a disordered state of the general health, though he thinks we are premature in referring it *exclusively* to the digestive organs.

"It has been well observed by Mr. Abernethy, that a disordered state of the digestive organs, and a local disease in a remote part of the system, may be concomitant effects depending upon some unknown irritation, proceeding, perhaps, from the nervous system, and that the medicine, which appears to act beneficially on the local disease, through the medium of the digestive organs, may, in fact, but correct a more general derangement of the health, of which a disorder in the chylopoietic viscera is but one of the effects." P. 351.

We think this is good reasoning, and coincide with Dr. Crampton, that the practitioner who looks exclusively to the digestive organs, as the sources of all local diseases, will be disposed to trust too much to medicine, and too little to those other influences which act most beneficially upon the human constitution.

*Case 5.* A general officer returned in 1813 from the Peninsula, suffering from various dyspeptic symptoms, as constipation, loss of appetite, depression of spirits, and unconquerable languor. His principal complaint was severe cephalalgia at night. The forehead was found covered with flattish tumours of various sizes, painful to the touch, and hard as a bone. Five grains of blue pill at night; a bitter draught with sulphate of magnesia twice a day; regulated diet; tepid salt water bath thrice a week. In two months the general health was much improved, he had gained flesh, and the tumours had totally disappeared.

*Case* 6. A lady had a swelling on the fore part of the tibia of the left leg, two inches above the ankle joint, about three inches in length, not very prominent, and of its natural colour. The tumour was soft, elastic, as though there was a sub-periosteal fluid; slight surrounding œdema; much nocturnal pain, with afternoon remissions. After local treatment had failed, a full course of mercury was gone through without benefit. In summer her sufferings were somewhat mitigated, and she ventured on the cold bath, but winter renewed the pain. She now came under the care of Mr. Todd and Dr. Crampton. In the previous treatment, local blood-letting had been omitted. Six leeches, therefore, were ordered to the tumour every evening; and during the day a camphorated evaporating lotion to the swelling. Attention to the state of the bowels. This plan failed, and an incision, the full length of the swelling, was made completely to the bone. Much blood, but no matter was discharged. The integuments were very much thickened, and the parts close to the bone had acquired a ligamentary firmness. No retraction of the edges of the cut. The case was bettered, but, as far as could be learnt, was not cured.

The following are the practical conclusions, which we shall give in Dr. Crampton's own words.

"1st. Inflammation of the periosteum, unconnected with specific disease, is an affection of very frequent occurrence. It often occupies the seat of the true venereal node, from which it can alone be distinguished by an accurate investigation of all the circumstances of the case.

"2nd. In the *acute form* of the disease recourse should be had to the means, both local and constitutional, which have the most decisive effects in discussing inflammation. Should these fail to procure relief, I believe there can be no doubt of the propriety of dividing the inflamed portion of periosteum through its whole length down to the bone.

"3rd. The same treatment is applicable to the *chronic form* of periostitis, with this difference, that in its early stages the disease may frequently be acted upon beneficially, through the medium of the constitution.

"4th. In the constitutional treatment, as our attention is to be directed to the improvement of the general health, our views should not be confined to the correcting a disordered state of the digestive organs, whether real or suspected. The constitution should also have the benefit of those influences which act most beneficially upon it; and of these I believe country air and sea-bathing are justly esteemed most powerful. Sarsaparilla, in whatever way it may act, is often eminently beneficial in cachectic habits, and particularly in those which have been injured by protracted courses of mercury. When chronic periostitis occurs in such habits, the compound decoction, or the syrup of the sarsaparilla may, in general, be given with considerable advantage."

We presume that we need not apologize for the length of this analysis, since the subject is very interesting in itself, and little treated of by systematic writers. The paper forms a good companion to that on exostosis by our great English surgeon, and certainly does not derogate from the well-earned reputation which Dr. Crampton has long and deservedly enjoyed.

